# Genomic and Protein Structural Maps of Adaptive Evolution of Human Influenza A Virus to Increased Virulence in the Mouse

**DOI:** 10.1371/journal.pone.0021740

**Published:** 2011-06-30

**Authors:** Jihui Ping, Liya Keleta, Nicole E. Forbes, Samar Dankar, William Stecho, Shaun Tyler, Yan Zhou, Lorne Babiuk, Hana Weingartl, Rebecca A. Halpin, Alex Boyne, Jayati Bera, Jessicah Hostetler, Nadia B. Fedorova, Katie Proudfoot, Dan A. Katzel, Tim B. Stockwell, Elodie Ghedin, David J. Spiro, Earl G. Brown

**Affiliations:** 1 Department of Biochemistry, Microbiology and Immunology, Faculty of Medicine, University of Ottawa, Ottawa, Ontario, Canada; 2 Emerging Pathogens Research Centre, University of Ottawa, Ottawa, Ontario, Canada; 3 National Microbiology Laboratory, Canadian Science Centre for Human and Animal Health, Public Health Agency of Canada, Winnipeg, Manitoba, Canada; 4 Vaccine and Infectious Disease Organization, University of Saskatchewan, Saskatoon, Saskatchewan, Canada; 5 National Centre for Foreign Animal Disease, Canadian Food Inspection Agency, Winnipeg, Manitoba, Canada; 6 Viral Genomics Group, J. Craig Venter Institute, Rockville, Maryland, United States of America; 7 Canadian Institutes of Health Research (CIHR) Canadian Influenza Pathogenesis Team, University of Ottawa, Ottawa, Ontario, Canada; 8 Center for Vaccine Research, Department of Computational and Systems Biology, University of Pittsburgh School of Medicine, Pittsburgh, Pennsylvania, United States of America; 9 Viral Genomics Group, J. Craig Venter Institute, Rockville, Maryland, United States of America; 10 Canadian Institutes of Health Research (CIHR) Canadian Influenza Pathogenesis Team, University of Ottawa, Ottawa, Ontario, Canada; University of Nebraska – Lincoln, United States of America

## Abstract

Adaptive evolution is characterized by positive and parallel, or repeated selection of mutations. Mouse adaptation of influenza A virus (IAV) produces virulent mutants that demonstrate positive and parallel evolution of mutations in the hemagglutinin (HA) receptor and non-structural protein 1 (NS1) interferon antagonist genes. We now present a genomic analysis of all 11 genes of 39 mouse adapted IAV variants from 10 replicate adaptation experiments. Mutations were mapped on the primary and structural maps of each protein and specific mutations were validated with respect to virulence, replication, and RNA polymerase activity. Mouse adapted (MA) variants obtained after 12 or 20–21 serial infections acquired on average 5.8 and 7.9 nonsynonymous mutations per genome of 11 genes, respectively. Among a total of 115 nonsynonymous mutations, 51 demonstrated properties of natural selection including 27 parallel mutations. The greatest degree of parallel evolution occurred in the HA receptor and ribonucleocapsid components, polymerase subunits (PB1, PB2, PA) and NP. Mutations occurred in host nuclear trafficking factor binding sites as well as sites of virus-virus protein subunit interaction for NP, NS1, HA and NA proteins. Adaptive regions included cap binding and endonuclease domains in the PB2 and PA polymerase subunits. Four mutations in NS1 resulted in loss of binding to the host cleavage and polyadenylation specificity factor (CPSF30) suggesting that a reduction in inhibition of host gene expression was being selected. The most prevalent mutations in PB2 and NP were shown to increase virulence but differed in their ability to enhance replication and demonstrated epistatic effects. Several positively selected RNA polymerase mutations demonstrated increased virulence associated with >300% enhanced polymerase activity. Adaptive mutations that control host range and virulence were identified by their repeated selection to comprise a defined model for studying IAV evolution to increased virulence in the mouse.

## Introduction

The mutational basis for the control of host switching (host-specific infection) and virulence (disease severity) in influenza A viruses (IAV) or their interrelationship is poorly understood [1]–[4] and the identification of genetic markers of host adaptation is the subject of much debate [5]. The existing knowledge of the evolution of virulence and host switching in IAV is incomplete as recently demonstrated by the introduction of a novel H1N1 IAV from swine into humans without the genetic markers associated with virulence and interspecies transmission [6]–[8]. Because virulence in IAV is controlled by mutations in multiple genes (see below) and novel virulent IAV rarely possess the same genetic markers, it is apparent that there are multiple genetic pathways for virulence and host-switching. Fundamental questions remain about the IAV adaptive mutations that modulate infection and disease, such as their identity, number, and repeatability of occurrence. Experimental studies of mouse adaptation (MA) of IAV identify parallel adaptive mutations that involve the repeated selection of mutation sites in HA and NS1genes among viruses from independent MA experiments [9], [10]. Parallel evolution is characteristic of drug resistance and is increasingly being observed among organisms that have evolved common traits ([11]–[13]. We now extend these studies of parallel evolution by performing full genomic sequence analysis of MA variants with mapping of all 11 viral proteins listed in Table 1. However, the links between IAV evolution, adaptation and virulence have yet to be elucidated.

**Table 1:** Influenza A virus genome structure and function.

genome segment	gene^a^	length (ntd)^b^	length (aa)	location in virion	functions^c^
1	PB2	2341	759	internal	transcription/capping/replication
2	PB1	2341	757	internal	transcription/replication
2	PB1-F2		91	nonstructural (cellular)	apoptosis
3	PA	2233	716	internal	transcription/replication
4	HA	1765	565	transmembrane	receptor/uncoating
5	NP	1565	498	internal	RNA synthesis
6	NA	1467	469	transmembrane	release
7	M1	1027	252	internal	assembly/regulation
	M2		97	transmembrane	uncoating
8	NS1	890	237	nonstructural (cellular)	IFN antagonist
	NEP		121	internal	nuclear export
all	all	13629	4562	na^d^	na

Specific values are for A/Hong Kong/1/68(H3N2).

a PB2 (polymerase subunit basic 2); PB1 (polymerase subunit basic 1), PB1-F2 (PB1-frame 2); PA (polymerase subunit acidic); HA (hemagglutinin); NP (nucleocapsid); NA (neuraminidase); M1 (matrix); M2 (M2); nonstructural 1 (NS1); nuclear export protein (NEP).

b nucleotide (ntd).

c details from reference[14].

d not-applicable.

IAV are enveloped with genomes composed of 8 negative sense RNA segments encoding 11 proteins (Table 1). IAV replication requires an ability to overcome host resistance and establish a productive infection that is achieved by entering cells to express genes that function to replicate and assemble genomes into virus particles. Replication also entails extensive interactions among viral proteins as well as host-factors [14]. Genome wide screens have identified 1,449 host proteins that are required for IAV replication [15] and a recent protein interaction analysis has identified 87 virus-host and 31 virus-virus protein interactions [16], however the binding sites of only a minority of these interactions are known [17]–[19]. Because replication occurs in the nucleus, the sites of interaction with host nuclear import and export proteins have been mapped for viral proteins involved in replication (reviewed [20]). It is generally assumed that adaptation of IAV to a non-permissive host involves mutations that overcome deficits in interaction with host factors to restore host factor binding such as seen for HA receptor binding to specific host sialic acids [10], [21]. Alternatively mutations affecting virus protein subunit interactions or functions such as HA fusion may also compensate for deficits in replication [10], [21].

Mammalian IAV species originate from the migratory aquatic bird reservoir of avian influenza viruses through processes that include reassortment of genome segments and adaptation of constituent genes [22]. Avian IAV species are typically non-pathogenic however they can evolve to become highly pathogenic strains that cause fatal infections in specific avian species [22], with some causing fatal infections in humans as seen for the 2003 H5N1lineage [23], [24]. The genetic basis for pathogenicity and host range has been extensively studied for 1918 H1N1 pandemic [25]–[28] and 1997 avian H5N1 viruses that are virulent for both humans and mice (with mouse LD_50_ values of ≤10^3.5^ pfu, [29], [30]). Genetic analyses in mice and human cells have identified roles in pathogenesis for HA, polymerase, PB1-F2 and NS1 genes in both 1918 H1N1 and H5N1 viruses (reviewed in [31]). The mutational basis for the virulence properties of most of these genes remains unknown except for sites in H5N1 HA [32] and NS1 [33], [34] as well as the PB1-F2 gene of both viruses [35], [36].

Adaptive evolutionary theory states that phenotypic variation and speciation is explained by the selection of biological variants that function to increase replicative fitness [37]. However a complete molecular theory of adaptation is still in development (reviewed by Orr [38]). Experimental studies of adaptation and variation demonstrate that large phenotypic changes involve the selection of a small number of mutations with those with the greatest effect selected first [39]. Recent genetic studies of bacteriophage host-range and virulence have demonstrated the repeated selection of identical or parallel adaptive mutations for 50% of amino acid (aa) substitutions among independent experiments [40]–[43]. Parallel evolution constitutes strong evidence of natural selection as characterized for drug resistant mutants [11], [12], [44].

Although phylogenic studies of humans and canine IAV show abundant variation, evidence of positive selection is generally lacking with nonsynonymous to synonymous mutation ratios (dN/dS) of <1 demonstrating stochastic variation [45]–[47]. However influenza viruses demonstrate both parallel and positive Darwinian evolution for mutations selected with neutralizing monoclonal antibodies [48], [49]. Antibody escape mutants are present at the rate of 1 per 1-3×10^5^ infectious viruses because populations of this size possess all single nucleotide polymorphisms (40,887 SNP's, see methods). In addition parallel evolution of drug resistance occurs in the M2 ion channel and neuraminidase (NA) where S31N and H274Y mutations are diagnostic of adamantane [50] and NA inhibitor resistance, respectively [51]–[53].

Although IAV are host restricted such that high dose intranasal infection of mice with human IAV does not typically result in disease, virulent MA variants that cause primary viral pneumonia at low dosage can be selected by serial mouse-lung passage (reviewed [54]). The mouse model has been shown to be relevant for the genetic analysis of pathogenesis of avian and mammalian IAV [55], [56]. We and others have demonstrated that adaptation to increased virulence in the mouse is associated with mutations that increase fitness and replication in virulent mouse-adapted variants [57]–[70] including the 2009 pandemic H1N1 strain [71]–[73]} (Table S1). These studies have generally identified polymerase and HA mutations as the most prominent aspect of adaptation to increased virulence, but have identified very few adaptive mutations in the smallest genes (NP, NA, M1/2, NS1/NEP) (see Table S1).

Mouse adaptation of A/HK/1/68 (H3N2) (HK-wt) by 20 serial passages in mouse lungs increased virulence by >10^5^ fold (reduction in LD_50_ from >10^7.7^ to 10^2.7^ plaque forming units (pfu)) [59]. Significantly, on initial sequence assessment of 3 HK-MA genomic clones plus M1/2 and NS1/NEP genes of 9 of 12 clones, we observed that 11 of 14 mutations were present in multiple clones and therefore under positive selection [59]. Subsequent HA gene sequence analysis of 42 mouse-adapted variants from 11 independent replicate mouse serial passage experiments identified 25 amino acid (aa) mutations with 4 sites demonstrating parallel evolution [10]. The observation of multiple MA variants with the same mutation in a group of 3–6 isolates, or alternately the same mutation in 2 virus isolates in independent mouse-adapted populations, cannot be explained by random chance and thus constitutes evidence of positive selection (P≤2×10^−8^ and P≤6×10^−7^, respectively, see methods). Sequence analysis of the NS1 gene of 42 MA variants of A/HK/1/68 identified 11 mutations with parallel evolution detected at position 106 (M106I and M106V) [9]. The parallel mutations selected in HA and NS were adaptive; increasing virulence and growth in mice when introduced into the HK-wt genome [9], [10]. The NS1 F103L and M106I mutations had also been selected in the A/HK/156/1997(H5N1) NS1 gene, where both mutations were shown to be required for the virulence property of this gene in reverse genetics studies [9]. Furthermore, reverse genetics studies of pathogenesis of the A/HK/1/68-MA (H3N2) and A/FM/1/47-MA (H1N1) variants, derived by serial high dose infection, showed that all of the mutant genome segments functioned to increase virulence in the mouse model [58], [74].

In this paper we extend our previous studies of HK-wt mouse adaptation by performing full genome sequencing of all 10 parental and 39 MA variants derived from 10 independent mouse-adaptation experiments in order to derive genetic and protein structural maps of adaptation to high virulence in the mouse. We identified adaptive regions within individual viral genes that included multiple instances of positive selection and parallel evolution.

## Results

### Assessment of adaptive evolution after 12 and 20 mouse-lung passages

We determined the nucleotide sequence of the genomes of 12 clonal isolates of HK-MA virulent variants that had been previously obtained after 12 and 20 serial mouse infections but only partially sequenced [59]. Sequence analysis of 6 clones derived after passage 12 showed that each clone acquired 4 to 7 (average 5.8) nonsynonymous mutations per genome (Table 2 and 3) that were responsible for their adaptation to increased virulence, (LD_50_ values of 10^5.4^ to 10^3.6^ pfu from [59] relative to >10^7.7^ pfu for HK-wt, (Table S2)). After 20 passages the average number of nonsynonymous mutations of 6 clones was increased to an average 8.8 per genome (Table 2 and 3) with LD_50_ values from 10^4.2^ to 10^2.6^ pfu [59] (Table S2). Comparisons of the individual mutations in each virus demonstrated more genetic heterogeneity at passage 12 than 20, with a trend to increased fixation of mutations within viral populations with increasing passage number (Table 2). Novel mutations were also selected at passage 20, including mutations in PB2, NP, M1, M2, NS1, and NEP (Table 2). An increased accumulation of mutations and virulence was observed with increasing numbers of cycle of serial mouse infections. The MA populations were under strong selection as evidenced by high nonsynonymous to synonymous (dN/dS) ratios for each virus; with an average of 2.8 for both the passage 12 and 20 virus groups (Table 3). Because we saw a greater selection of mutations in the smaller genes by passage 20 our subsequent mouse adaptation experiments employed 21 passages.

**Table 2:** Mutations in genomes of mouse-adapted variants obtained after 12 and 20 mouse-lung passages relative to HK-wt.

		HKMA12 clones and mutations						HKMA20 clones and mutations						
gene	HK-wt^a^	12^b^	12A	12B	12C	12D	12E	20^b^	20A	20B	20C^a^	20D	20E	positive selection
PB2	D701	N	N	N	N	N	N	N	N	N	N	N	N	yes
	D740	•	•	•	•	•	•	•	N	•	•	•	•	nd
PB1	R190	•	•	•	•	•	•	K	•	•	•	•	•	nd
	K578	•	T	•	•	T	•	T	•	•	•	T	•	yes
PA	D27	G	•	•	G	•	•	•	•	•	•	•	•	yes
	Q556	•	•	•	•	•	R	R	R	R	R	R	R	yes
	E610	•	•	G	•	•	•	•	•	•	•	•	•	nd
	K673	•	•	R	•	•	•	•	•	•	•	•	•	nd
HA^c^	D2^1^	•	Y	•	•	•	•	•	•	•	•	•	•	nd
	P162^1^	•	•	L	•	•	•	•	•	•	•	•	•	nd
	G218^1^	W	•	•	W	W	W	W	W	W	W	W	W	yes
	N246^1^	•	D	•	•	•	•	•	•	•	•	•	•	nd
	N154^2^	•	•	•	•	•	•	•	•	•	•	S	•	yes
	T156^2^	N	•	•	N	N	•	N	N	N	N	•	N	yes
	D158^2^	•	•	N	•	•	•	•	•	•	•	•	•	nd
NP	D34	N	N	N	N	N	N	N	N	N	N	N	N	yes
	D480	•	•	•	•	•	•	•	•	N	N	N	•	yes
NA	A110	•	•	•	•	•	•	•	•	•	•	V	•	nd
	P468	H	H	H	H	•	•	H	•	H	H	•	H	yes
M1^b^	D232	•	•	•	•	•	•	N	N	•	•	•	•	yes
M2^b^	D44	•	•	•	•	•	•	•	•	N	N	.	N	yes
NS1^a^	V23	•	•	•	•	A	•	A	A	•	•	•	•	yes
	F103	•	•	•	•	•	•	•	•	L	L	L	•	yes
NEP^a^	K88	•	•	•	•	•	•	•	•	R	•	•	•	nd

a previously independently sequenced in [59].

b partially independently sequenced as reported in [59].

c mutation found in multiple clones is indicated (yes) versus not-detected (nd).

d independently sequenced in [10].

e HA mutations in the HA1 and HA2 subunits are indicated with superscripts 1 and 2 respectively.

f identity to HK-wt amino acids is indicated by dots.

g same loss of glycosylation as HA T156^2^N.

**Table 3:** Assessment of nonsynonymous (dN) versus synonymous (dS) nucleotide changes (ntd), are shown for HKMA12 and HKMA20 variants relative to HK-wt.

		HKMA12 clones						HKMA20 clones						
genome values	HK-wt	12	12A	12B	12C	12D	12E	20	20A	20B	20C	20D	20E	average
total ntd change	0	9	10	9	9	7	6	14	12	14	12	11	10	10.3
total dN	0	6	6	7	6	6	4	10	8	10	9	9	7	7.3
total dS	0	3	4	2	3	1	2	4	4	4	3	2	3	2.9
dN/dS	0	2.0	1.5	3.5	2.0	6.0	2.0	2.5	2.0	2.5	3.0	4.5	2.3	2.8

### Independent MA experiments demonstrate parallel evolution

**Figure 1:**
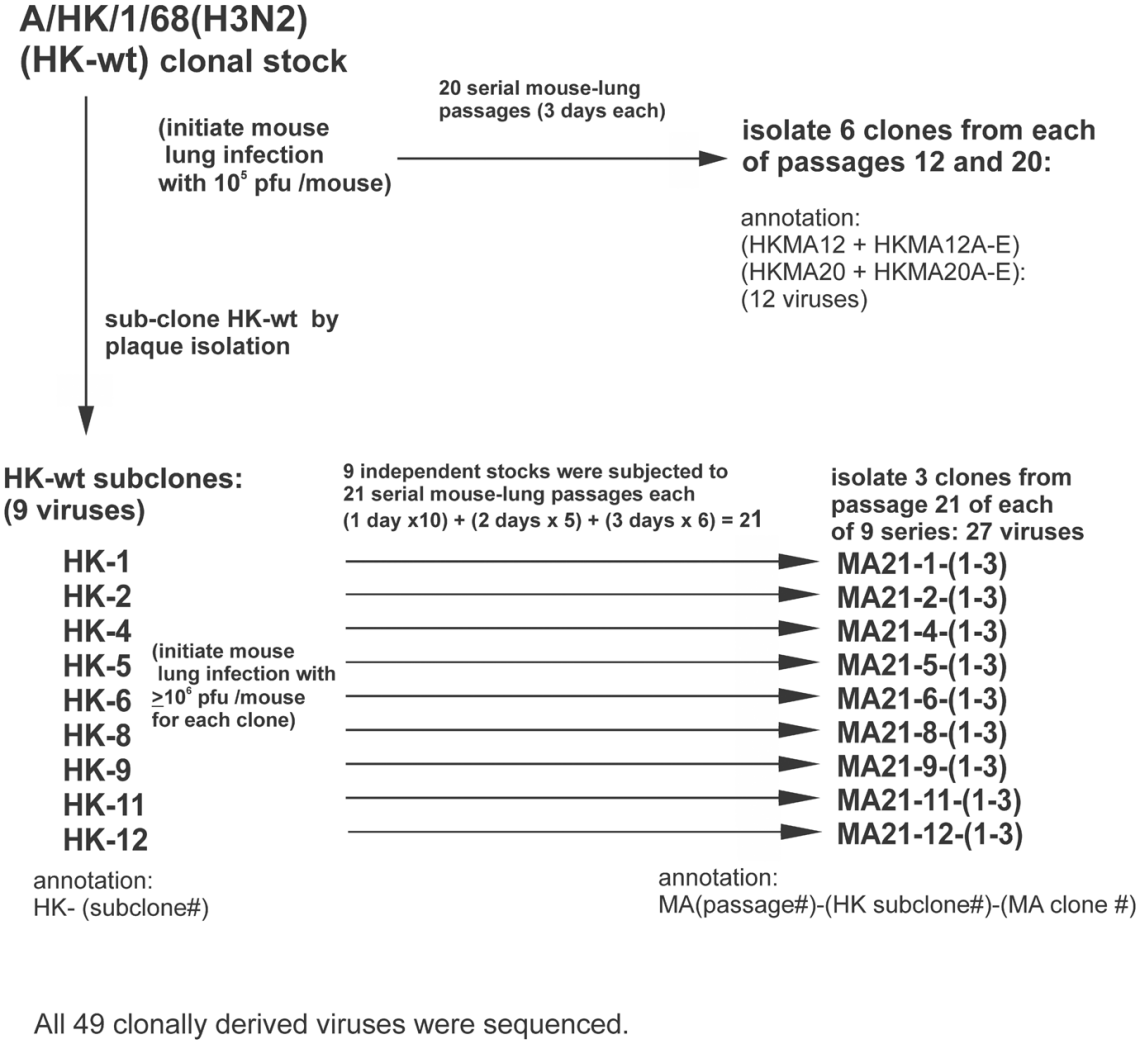
Experimental design of parallel studies of mouse adaptation. The parental strain of A/Hong Kong/1/68 (HK-wt) was clonally derived on MDCK cells and grown in chicken embryos before dilution in PBS to 1×10^5^ pfu/ 0.05 mL to initiate serial mouse passage; followed by 20 serial passages with 6 clones derived by plaque isolation from the passage 12 and 20 populations on MDCK cells. Replicate stocks of HK-wt (HK-#) were generated from individual infectious virions of HK-wt by plaque isolation. Each subclone was amplified in eggs and used without dilution to infect mice (≥1×10^6^ pfu/0.05 mL for each mouse) to initiate 9 parallel MA series as indicated in methods before isolating 3 clones from each of the passage 21 populations.

Fig. 1 illustrates the strategy used for performing multiple independent MA experiments. Each of 9 HK-wt clones was subjected to a total of 21 mouse passages before isolating 3 MA clones from each passage 21 population, that were then annotated as HKMA21-population #-clone# (Fig. 1). Viral stocks of the 9 HK-wt subclones provided viral populations that originated from individual virus particles (HK-wt subclones) and thus the selection of mutations in these populations constitutes independent events relative to those mutations characterized in the passage HKMA12 and 20 populations (Table 2).

The genomes of each HK-wt subclone as well as each of the 27 HKMA21 variants was subjected to full genome sequencing to identify mutations selected on mouse-adaptation (Genbank numbers in Table S9). Only one coding mutation and 8 synonymous mutations were found among the 9 subclones of HK-wt (Table S3, S4, S5, S6, S7, S8) indicating that each genome possessed an average of 1 single nucleotide polymorphism that defined the parental sequence used to initiate infections in the 9 independent MA series. Comparison of the gene sequences of 27 HKMA21 clones with their corresponding HK-wt clones identified an average of 7.7 non-synonymous mutations per MA genome (Table S2). Of the 429 sequenced MA genes (from 39 MA viruses) most possessed 0 or 1 mutations (45 and 42% respectively) with 2 or 3 mutations in 11 and 1.4% respectively (Table S2). The HA and PB2 genes acquired the most mutations on average, 1.54 and 1.38 respectively, with most mutations selected in the ribonucleocapsid complex of genes (PB2+PB1+PA+NP) that possessed an average of 4.33 mutations per genome, relative to the remainder of the genome (HA+NA+M1+M2+NS1+NEP+PB1-F2 genes) that possessed an average of 3.23 mutations per genome (Table S2). The LD_50_ values for 7 of the 27 MA21 variants ranged from 10^1.1^ to 10^6.5^ pfu (Table S2).

### PB2 protein

**Figure 2:**
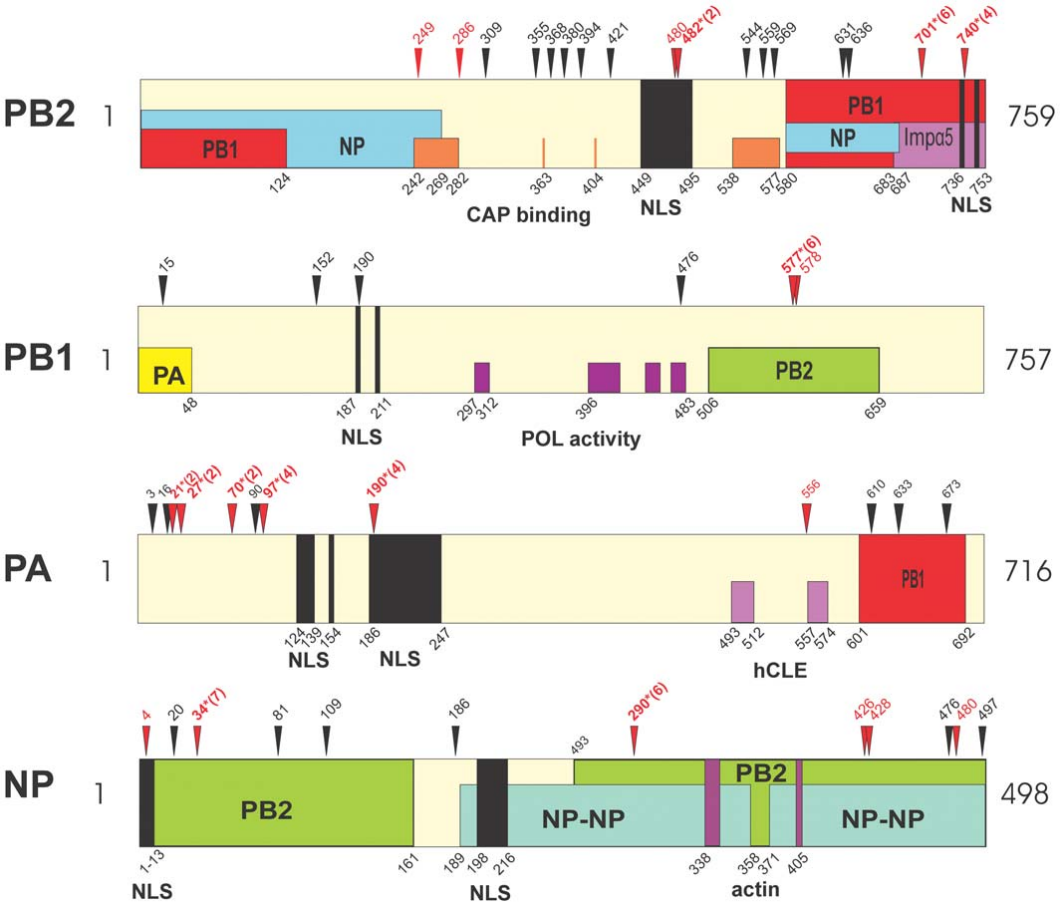
Mouse adaptive mutations on the primary structural maps of PB2, PB1, PA and NP proteins. The amino acid location of mutations are numbered and indicated with arrowheads on the linear sequence, sites of positive selection are shown red and parallel mutations are additionally indicated with an asterisk and the number of populations in parenthesis. The locations of regions of interaction, or functions are indicated with rectangles and are labeled with respect to interacting viral proteins as indicated in Methods. Nuclear localization signals (NLS) are in black, and host protein sites are indicated for PB2, PA and NP; PB1 polymerase activity regions are in purple and PB2 cap binding regions are in orange. hCLE, the human transcription factor is positioned In PA according to [124] The following mutations were mapped previously: PB2 D701N, PA Q556R, NP D34N, and NP D480N[59].

**Figure 3:**
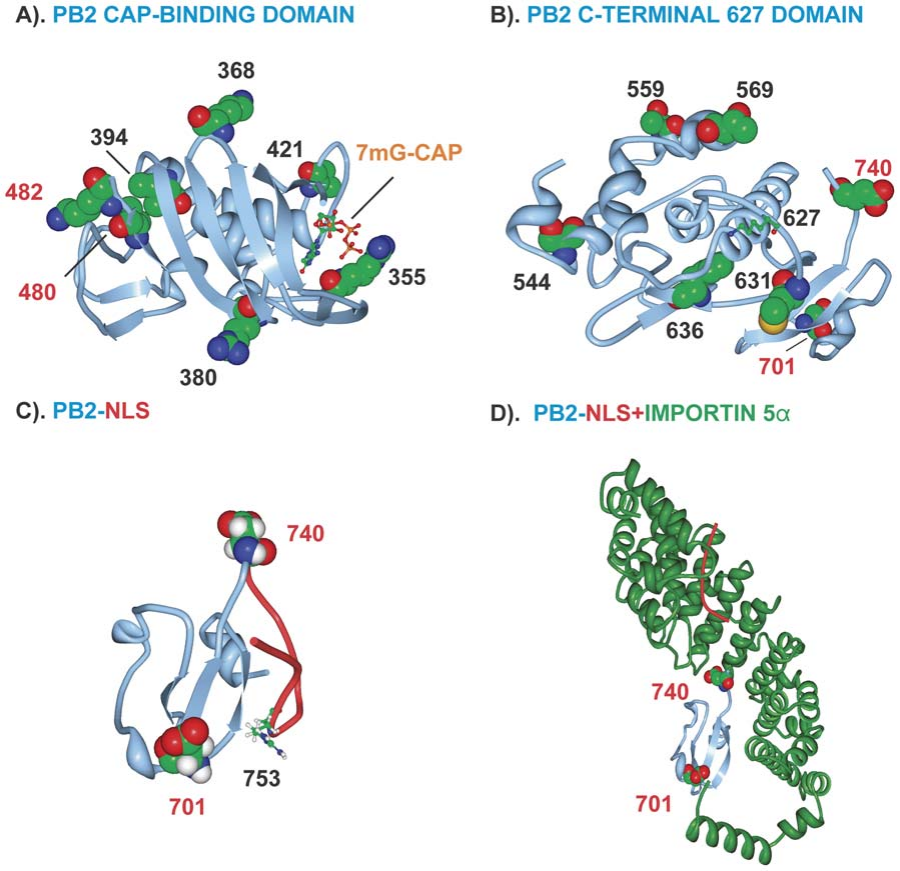
Mouse adaptive mutations on PB2 three dimensional maps. Mutation sites are shown on ribbon structures of PB2 protein with space filling models of amino acids numbered in black for mutations found once, or red for positively selected mutations. (A) PB2 cap binding domain bound to m7GTP (in stick image); (B) PB2 C-terminal domain with the 627 site shown in stick image. (C) PB2 NLS with R753 (stick image) that forms a salt bridge with D701, NLS in red; (D) PB2 NLS in complex with human importin α5; NLS in red.

Genome segments of each MA21 derivative were aligned with respect to the coding sequence for each parental strain to identify mutations (Tables S3, S4, S5, S6, S7, S8). Sequence comparison of the MA PB2 genes showed that 100% of the MA strains possessed mutations with 1 to 3 coding substitutions each (Table 2 and S3). Parallel evolution was seen for the D701N, D740N and K482R mutations that were obtained in 6, 4, and 2 populations respectively. PB2 D701N and D740N were the most commonly selected, found in 25 and 7 of 39 clones, respectively (Table 2 and S3). Positive selection as evident by the isolation of multiple mutants with the same mutation from the same population was also seen for 3 other mutations: V480I, E249G and S286G (shown in red in Table S3). PB2 mutations clustered in regions on both the primary (Fig. 2) and 3D structure maps (Fig. 3), involving the nuclear localization signal (NLS) and cap binding domains. PB2 mutations between position 249 and 569 reside in the host 7methyl guanosine cap binding domain with the R355M and V421 mutations in contact with the cap phosphate as well as in a solvent exposed loop of PB2, respectively [75] (Fig. 3A). PB2 mutations between 554 and 740 surround the 627 site in the C-terminal domain (Fig. 3B). The PB2 D701N mutation disrupts a salt bridge with R753 that sequesters the nuclear localization signal to result in NLS release (Fig. 3C). In addition D701N and D740N mutations occurred in the NLS domain that binds human importin 5α (Fig. 3D) [76].

### PB1 protein

**Figure 4 pone-0021740-g004:**
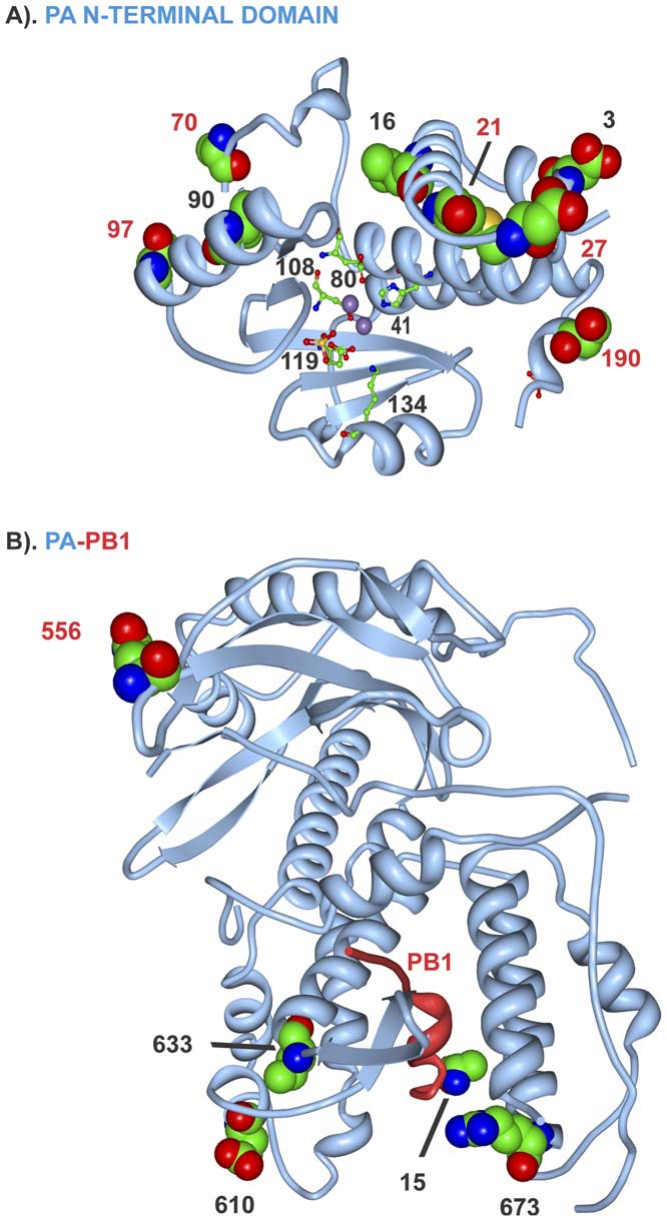
Mouse adaptive mutations on PA and PB1 three dimensional maps. Images are shown as in Fig. 3. A) PA N-terminal domain with nuclease site active residues, H41, E80, D108, E119 and K134 shown in stick diagram; manganese ions are shown with purple spheres. (B) PA (blue) PB1 (red) complex; PA amino acids 1–14 are in direct contact with PB2[125].

The PB1 protein was more genetically conserved than PB2 with 12 of 27 MA21 clones (44%) possessing mutations; all were single mutations except one double mutation (Table S4). Parallel evolution was seen at PB1 aa position 577, with 10 clones in 6 MA21 populations possessing 3 alternative mutations (K577E, K577M or K577Q). The 577 residue is adjacent to the K578T mutation selected in the HKMA12+20 population (Table 2) thus defining a pair of adjacent adaptive sites that map to the center of the PB2 binding site (Fig. 2). PB1 mutation R190K resides in a nuclear localization site and a mutation at N476S maps to a site involved in RNA polymerase activity (Fig. 2). The PB1 Q15H mutation maps to the amino terminal PA binding region (Fig. 2) adjacent to 14 terminal amino acids that insert into the PA binding pocket of the PB1-PA co-crystal (Fig. 4B).

### PA protein

The PA protein was highly adaptive with 24 of 27 MA21 clones (89%) possessing 1 or 2 mutations (Table S4). Considering all PA mutations, parallel evolution was seen for M21I, D27G, A70V, T97I and S190F/T mutations (Table 2 and S4). These mutations localized to the PA amino-terminal domain comprised of aa 1–209 (Fig. 2 and 4A) that is involved in multiple functions, including transcription, replication, RNA endonuclease, and cap binding [77]. These parallel mutations as well as D3G, L16I, M21I and V90I cluster on two surfaces of the PA amino terminal domain 3D structure (Fig. 4A) adjacent to the nuclease active site residues (H41, E80, D108, E119, K134 in stick model with 2 Mn^++^ ions in Fig. 4A). The S190F and S190T mutations reside in one of the NLSs and the 556 site was adjacent to the hCLE host transcription factor binding site [19] (Fig. 2). All 3 C-terminal mutations, E610G, I633V, and K673R, (Table 2 and S4) mapped to sites in the PB1 binding region (Fig. 2).

### NP protein

**Figure 5:**
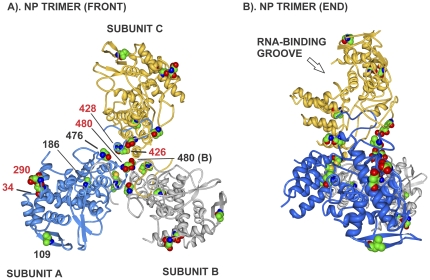
Mouse adaptive mutations on NP protein trimer three dimensional maps. A). The mutations are shown on the asymmetric NP trimer of subunits A (blue), B (grey) and C (gold). Mutations are shown in numbered space filling models on the ribbon backbone of subunit A with the exception to position 480 that is shown on both subunits A and B. Mutations are numbered and shown as described in Fig. 3. Mutation sites in contact with NP A and B subunits in the oligomer are: M426(B) to M448+E449(A); A428(B) to R261(A), V476(B) to D482+S483(A); and D480(A) to M481(B). B). Side view of trimmer showing the clustering of mutation on alternate faces that define adaptive domains.

The NP protein was mutated in 100% of MA clones with parallel evolution seen for D34N and D290N/E mutations in 8 of 9 HKMA21 populations and 85% of the variants (23 of 27), (Table 2 and S5). It appears that both mutation sites may affect similar functions because they are juxtaposed on the 3D map (Fig. 5), but reside in separate regions of primary structure that have been involved in PB2 interaction (Fig. 2). The carboxyl terminal mutations, M4261, A428T, V476A, D480N and D497N, map to overlapping NP and PB2 interaction regions (Fig. 2). Positions V476, and D480 of subunit A bind to subunit B in the NP-trimer complex and M426I and A428T are located in the tail loop that contacts adjacent NP molecules (Fig. 5A). The V186I mutation is located on the surface of the RNA binding groove (Fig. 5). The Q4K and Q20P mutations map to a NLS site binding region (Fig. 2) but were not resolved in the crystal structure (Fig. 5).

#### HA protein

**Figure 6:**
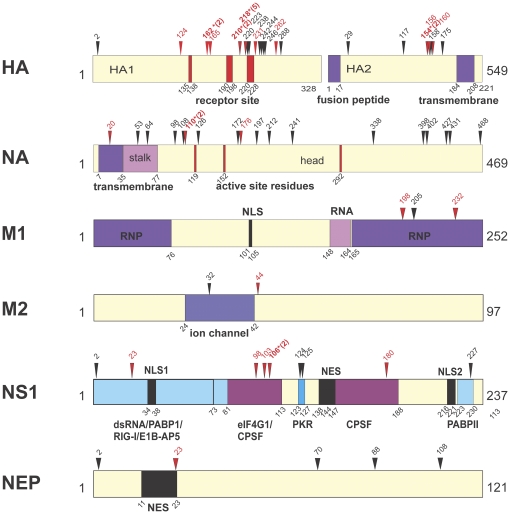
Mouse adaptive mutations on the primary structural maps of HA, NA, M1, M2, NS1 and NEP proteins. Mutations are shown as indicated for Figure 3. The locations of protein binding and active sites are indicated; RNP ribonucleocapsid protein; NLS nuclear localization signal; NES nuclear export signal; dsRNA double-stranded RNA (aa 1–73); PABPI poly-A binding protein 1; PABPII poly-A binding protein II; RIG-I retinoic acid inducible gene I; E1B-AP5, E1B associated protein 5; CPSF cleavage and polyadenylation specificity factor; eIF4GI eukaryotic initiation factor 4GI; and PKR protein kinase R. The following mutations were mapped previously: HA1 G218W, HA2 T156N, NA P468H, M1 D232N, NS1 F103L + V23A, NEP K88R [59].

**Figure 7 pone-0021740-g007:**
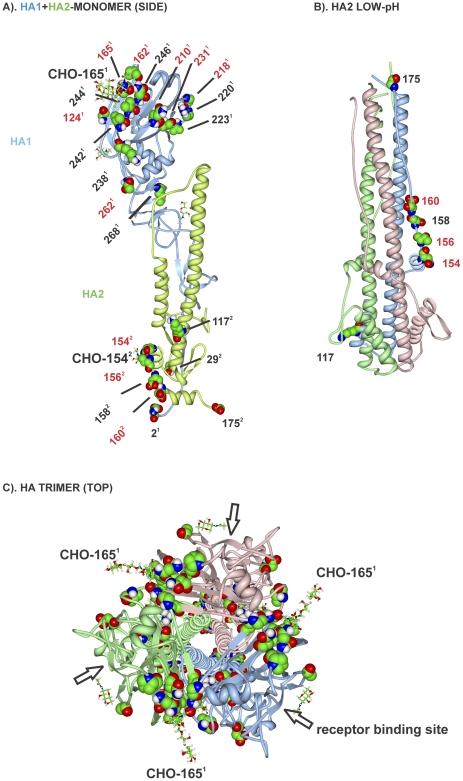
Mouse adaptive mutations in the crystal structure of the HA protein monomer, trimer and low-pH HA2 trimer. Mutations are shown as described in Fig. 3. (A) the side view of the HA monomer composed of HA1 and HA2 (indicated with superscripts 1 and 2) with carbohydrate side chains shown (CHO) in stick diagram is included here for reference (a similar but unglycosylated map that was generated from an independent sequence analysis has been published [10]); (B) the low pH form of HA2; and (C) the HA trimer, top view with receptor sites indicated with arrows.

The HA mutations from 42 HK MA variants have been independently sequenced and presented previously on the 3D map of the HA monomer [10], which we have now generated in modified form from independently derived sequence data (data of Table S1 shown in Fig. 6 and 7A). In addition, we present novel maps of the HA trimer and low pH form of the HA2 trimer (Fig. 7B and 7C, respectively) as well as the HA1 and HA2 primary sequence maps (Fig. 6). We observed a total of 25 HA gene mutations involving 37 of 39 HKMA clones (Table 2 and S6) that included 4 sites with parallel evolution (HA1 positions 162^1^, 210^1^, 218^1^, and HA2 154^2^) with 6 more showing positive selection (G124^1^D,N165^1^D, S231^1^N, T262^1^N, T156^2^N, and D160^2^N). The mutations were clustered in 2 regions of the primary and 3D maps. One region in HA1 (Fig. 6) defined a HA1-HA1 contact face adjacent to the receptor binding site and the 165^1^ glycosylation site (Fig. 7A and C). The second adaptive region was around the 154^2^ glycosylation site in the HA2 subunit that included mutations between positions 154 to 160 that aligned on a loop extending to the transmembrane region in the low pH form of HA2 (Fig. 6 and 7B).

### NA Protein

**Figure 8 pone-0021740-g008:**
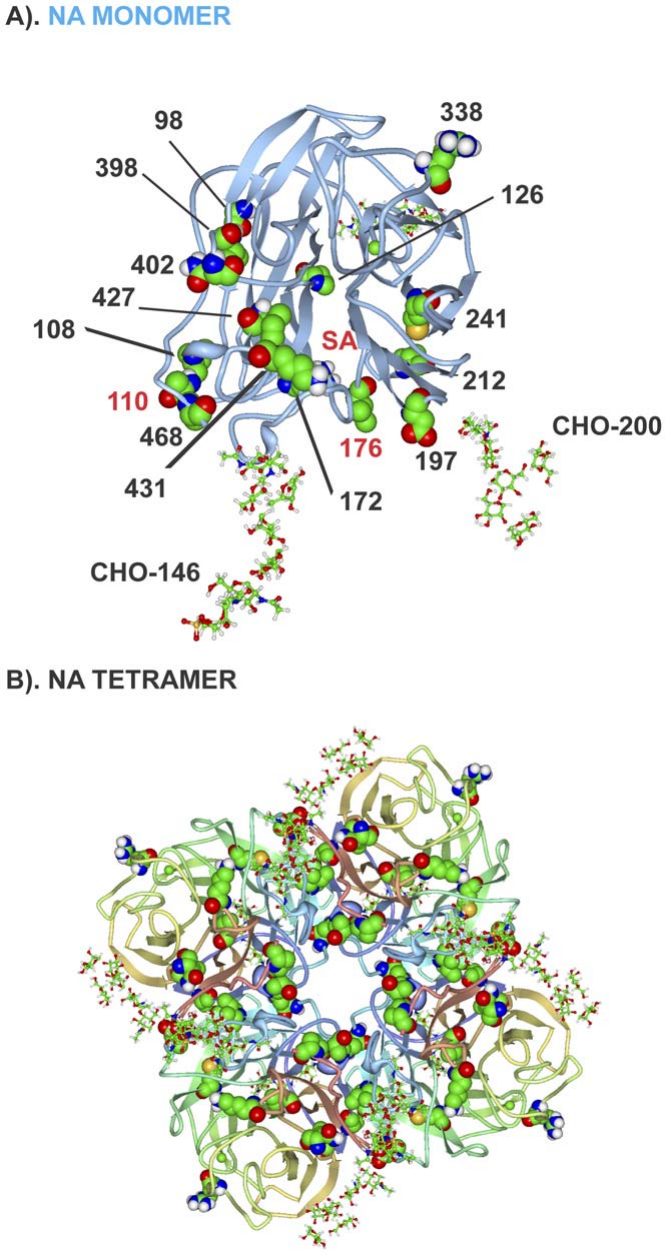
Mouse adaptive mutations on NA three dimensional maps. Mutations are shown as described in Fig. 3. (A) Mutations are shown in the NA monomer with receptor site indicated with SA and carbohydrate (CHO) in stick diagram; (B) the tetrameric form of NA.

The NA protein was mutated in 18 of 27 (67%) MA21 clones (Table S7 and 2). Parallel evolution was seen at position 110 (MA21-5-1 and MA20) and positive selection was observed for mutations at sites 20 and 176. The mutations formed 2 groups on the primary sequence map that were in the amino terminal half of the protein including the transmembrane domain, and in the C-terminal region (Fig. 6). On the 3D structural maps, the mutations primarily localized on the top surface around the sialic acid binding and glycosylation sites as well as contacts with adjacent NA monomers in the tetrameric structure (Fig. 8).

### M1 and M2 protein

**Figure 9 pone-0021740-g009:**
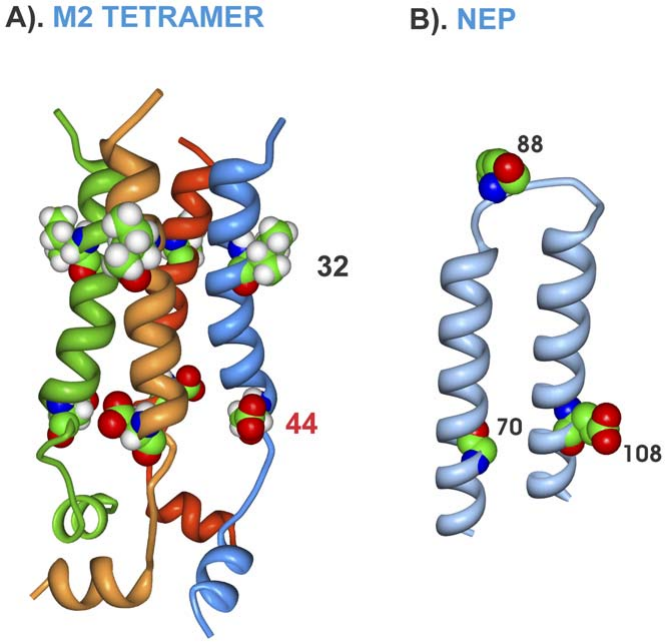
M2 tetramer and NEP three dimensional maps of mouse adaptive mutations. Mutations are shown as described in Fig. 3. (A) on the M2 tetramer and (B) the NEP protein.

M1 and M2 are overlapping genes encoded in different reading frames. Both were highly conserved among MA21 clones; with 3 and 4 variants, respectively (Table S8 and 2). M1 mutations showed positive selection for 2 of 3 sites in the C-terminal region of unsolved 3D structure between residues 198 and 232 in a region that has been shown to bind ribonucleoprotein (RNP) (Fig. 6). Only 3 mutations were observed in the M2 protein, at position 26 and 32 and the positively selected mutation at 44, all of which resided in or near the ion channel domain (Table 2 and S8; Fig. 9A). All 3 clones of the HKMA21-12 population possessed an M2 L26F mutation that was present in the HK-12 parental clone and thus was not selected during mouse passage (Table S8). The mutations at residues 32 and 44 were adjacent to the same residues of adjoining monomers in the tetrameric ion channel (Fig. 9A).

### NS1 and NEP protein

NS1 and NEP are also overlapping genes encoded in different reading frames but were more variable on mouse adaption than the M1 and M2 proteins. Four NEP mutants were observed among the MA21 clones and one in MA20B (Tables 2 and S8) that were distributed along the length of the primary structure with the positively selected S23P mutations occurring in the nuclear export signal site (Fig. 6). The NEP G70S and E108K mutations were adjacent to each other on the 3D structure suggesting that they may affect a similar function (Fig. 9B).

**Figure 10:**
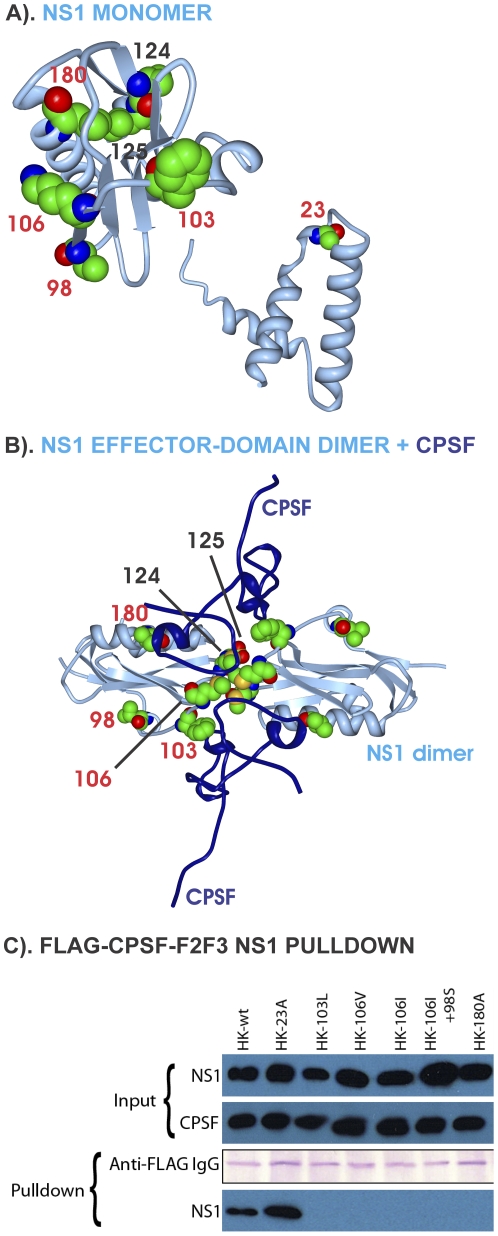
NS1 three dimensional maps of mouse adaptive mutations and effects on CPSF binding. Mutations are shown as numbered space filling images as described in Fig. 3. (A) in the NS1 monomer; (B) the NS1 dimer effector domain (grey) bound to 2 CPSF F2F3 fragments (dark blue). Amino acid NS1 106 of each monomer is in direct contact in the dimer. CPSF30-F2F3 is in direct contact with NS1 amino acids 103, 106 and 180. (C) Coimmunoprecipitation of HK-wt and V23A, F103L, M106V, M106I, M106I+L98S, and V180A mutant NS1 proteins with FLAG-CPSF30-F2/F3. Recombinant NS1 proteins (2.0 µg) were mixed with equivalent amounts of FLAG-tagged CPSF30-F2/F3 before blotting in parallel using anti-NS1 or anti-FLAG monoclonal antibody respectively to demonstrate the input. Pull down samples were blotted in side-by-side comparisons for immunoglobulin (as a loading standard) and NS1 protein to demonstrate association of NS1 with CPSF30-F2/F3.

The NS1 proteins possessed 8 mutations among 11 MA21 variants occurring as single or double mutations (Table S8) that have been reported previously but have not been mapped [9]. Among 10 mutations that included 2 mutations in MA20 viruses, parallel evolution was seen for M106I, and M106V and positive selection was seen for V23A, L98S, F103L, and V180A (Table 2 and S8). The two most adaptive regions encompassed the 98, 103 and 106 sites in the middle of the protein in binding regions of the eukaryotic translation initiation factor 4GI (eIF4GI) and the cleavage and polyadenylation factor 4 (CPSF30), in addition to the M124I and D125G mutations in the PKR binding site (Fig. 6). The D2N and V23A mutations resided in the RNA/PABP1/RIG-I/EIB-AP5 binding domain (Fig. 6) and mutations V180A and R227K were found in the CPSF30 binding and PABPII binding domains respectively (Fig. 6). Mapping the mutations on the 3D structure of the NS1 dimer in complex with the C-terminal CPSF30-F2F3 fragment showed that the 106 site of each NS1 monomer were in direct contact, and positions 103, 106, and 180 were in contact with CPSF30 (Fig. 10A and 10B). Each of the mutations in contact with CPSF30 resulted in a loss of CPSF30 binding in pull-down assays of recombinant NS1 proteins (Fig. 10C). Western blots of input levels of NS1 and CPSF30-F2F3 are shown relative to pull down levels of anti-FLAG IgG (Coomassie brilliant blue stained) and NS1 proteins (anti-NS1 western blot) where HK NS1-wt and the HK NS1-V23A both bound CPSF30-F2F3 and the F103L, M106V, M106I, M106I+L98S, and V180A did not bind CPSF30-F2F3 (Fig. 10C) (similar data were obtained with the full length CPSF30 protein (data not shown)). None of the recombinant NS1 proteins were immunoprecipitated from control pull-down assays that used empty vector transfected 293T cell lysates (data not shown). These data indicate that mouse-adapted NS1 mutations in the CPSF30 binding site decrease CPSF30 binding which would be predicted to reduce inhibition of mRNA processing [78].

### Validation of PB2, PA and NP mutations as virulence determinants in the mouse model

**Figure 11:**
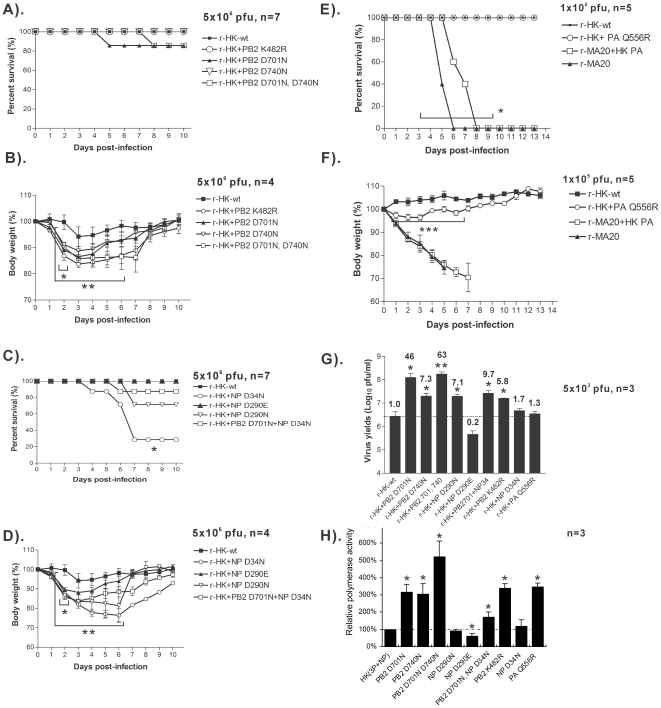
Assessment of the roles of PB2, PA and NP mutations in mouse models of virulence, replication and polymerase activity. Body weight and survival were monitored for groups of mice infected with recombinant HK viruses that possessed mouse adaptive mutations. (A and B), r-HK-wt and PB2 mutants, (K482R, D701N, D740N, D701N+D740N) were used to infect groups of 4 mice with 5×10^6^ pfu of virus for body weight loss and with n = 7 for survival. (C and D) r-HK-wt and mutant viruses with NP mutations D34N, D290N, D209E, or PB2 D701N+NP D34N were used as indicated to infect groups of 4 mice with 5×10^6^ pfu of virus and with n = 7 for survival. (E and F) r-HK and r-HK + MA20-PA (Q556R) as well as r-MA20 and r-MA20 + HK-wt-PA were used to infect groups of 5 CD-1 mice with 1×10^5^ pfu of each with monitoring of weight loss and mortality. Weight loss was assessed relative to r-HK-wt infections using the single sample t-test at day 2 pi or using the paired t-test for 2–6 dpi; time to death was significant as indicated using student's t-test, P<0.05 indicated with *. (G) Replication in mouse lungs was monitored 1 dpi after infection of groups of 3 mice with 5×10^3^ pfu each. The values are means of infections yields ±SD for groups of 3 mice. (H) Polymerase activity was measured in mouse B82 cells for each of the indicated mutations relative to luciferase minigenome expression via a mouse POL1 polymerase by HK-wt plasmids expressing PB1, PB2, PA and NP and firefly luciferase driven by the NP promoter. Values are means ±SD for n = 3 samples. Statistical significance relative to HK-wt at the P≤0.05 and P≤0.01 are indicated with * and ** respectively.

Recombinant HK-wt (rHK-wt) and mutant viruses that differed from HK-wt due to each of the parallel PB2 mutations, K482R, D701N, D740N, as well as D701N+D740N were generated using reverse genetics. We assessed the ability of each of these combinations of PB2 mutations to cause disease in groups of mice that had been infected with each virus and monitored for weight loss and lethality. Because the LD_50_ of HK-wt is >10^7.7^ pfu [59] increased mortality is not usually observed due to single additional mutations therefore increased disease severity is measurable by weight loss [74]. Although all mutations induced increased weight loss (P≤0.05 at day 2 post infection (pi) and P<0.01 by paired t-test from day 2 to 6 for all mutants), the greatest effect was seen for the K482R mutant. Only the D701N mutation on its own or in combination with D740N resulted in mortality (14% each), indicating that the LD_50_ of each mutant virus was >5×10^6^ pfu (Fig. 11A and B). The D701N + D740N mutations in combination resulted in more prolonged weight loss, than for each mutation in isolation (Fig. 11B).

Similar infections of mice with rHK viruses that possessed the parallel NP mutations D34N, D290N, D209E or PB2 D701N+NP D34N showed increased lethality relative to HK-wt for the D34N and D290N mutations (72% and 28% respectively). All mutants had an LD_50_>5×10^6^ pfu except for D34N (LD_50_ = 2.7×10^6^ pfu) that also caused a significantly reduced time to death relative to HK-wt (≤0.05 by t-test). All of these mutants enhanced disease severity as monitored by weight loss (P≤0.05 at day 2 pi and P<0.01 by paired t-test for days 2 to 6 for all mutants) (Fig. 11C and D). Lethality and weight loss (from day 4 to 10) was reduced for the PB2 D701N+NP D34N mutations relative to that of NP D34N suggesting gene interaction effects between these mutated NP and PB2 genes.

We also tested the effect of the PA Q556R mutation on the HK-wt backbone and the replacement of PA Q556R with HK-wt PA on the HKMA20 backbone. Infection of groups of 5 mice with 1×10^5^ pfu of each virus showed that the PA Q556R mutation resulted in significantly increased body weight loss (P≤0.001 by paired t-test) on the HK-wt backbone and a decreased time to death (P≤0.05 by t-test) in the MA20 virus relative to rMA20 + HK PA (Fig. 11E and F). The LD_50_ of r-MA20 + HK PA was 10^3.8^ pfu relative to the LD_50_ of 10^2.9^ pfu for r-MA20, indicating that the PA Q556R mutation increased virulence by 8 fold.

The effect of PB2, NP, and PA mutations on viral replication in mouse lung was also measured at 1 dpi relative to rHK-wt by plaque assay of lung homogenate for groups of 3 mice infected with 5×10^3^ pfu. Significantly increased yields ranging from 5.8 to 43 fold more than rHK-wt was seen for NP D290N and PB2 mutations (K482R, D740N, and D701N). NP mutations D34N, D209E, and PA Q556R did not significantly increase yields relative to rHK-wt at this time point (Fig. 11G) nor at 3 dpi (data not shown). Replication of the PB2 D701N+D740N was increased whereas the PB2 D701N+NP D34N mutant was decreased relative to PB2 D701N alone (Fig. 11G), which reflected the relative differences seen in virulence and/or body weight loss in mice infected with these viruses (Fig. 11A–D). The NP D34N and D290E mutations both increased disease severity but did not significantly enhance replication indicating that their increased virulence was replication-independent. Using reverse-genetics we have shown that each of the 7 tested PB2, NP and PA mutations increased virulence but that this was not proportional to replication in the mouse lung that demonstrated epistatic effects between PB2 and NP mutations.

The effect of PB2, PA, and NP mutations on RNA polymerase activity was measured using the luciferase minigenome assay in mouse B82 cells (Fig. 11H). The PB2 K482R, D701N, D740N, and PA Q556R, all increased polymerase activity by >300%. In addition the PB2 D701N + D740N mutations were additive to enhance activity by >500% and thus demonstrated their adaptive properties in mouse cells (Fig. 11H). The polymerase activities of the NP D290N and D34N mutations were reduced or not changed relative to HK-wt, respectively. Activity was reduced by half for NP D34N + PB2 D701N relative to the PB2 D701N mutation alone, demonstrating negative epistatic effects for both NP mutations.

Because the D34N mutation was the most highly selected NP mutation (Table 4) but reduced D701N polymerase activity we assessed the effect to this mutation in several combinations of polymerase mutations including those found in HKMA12 and HKM20 clones in both mouse and human cells (Table 2). The PB2 D701N, PB1 R190K+K578, PA Q556R, NP D34N and NP D34N+D480N mutations were included as controls. In mouse B82 cells, all individual mutant polymerase subunits, PB2 D701N, PB1 R190K+K578T, and PA Q556R (Fig. S1A bars a-f) as well as combinations significantly increased RNA polymerase activity by >250% of HK–wt activity (Fig. S1A bars i-m, HKMA12E bar j, HKMA20B, 20C and 20D bar m). The HKMA12A and 12D (PB2 D701N + PB1 K578T + NP D34N) increased activity by 648% (P≤0.05 by t-test) (Fig. S1A bar i). The PB1 K578T mutation increased polymerase activity but the R190K mutation did not and reduced activity when in combination with K578T indicating epistatic effects (Fig. S1A bar c,d,e). Assaying polymerase activity in human 293T cells showed increased activity to approximately half of the levels seen in the mouse cells for the PB2 D701N and PB1 K578T, but not PA Q556R mutation (Fig. S1B). The NP D34N mutation increased activity by about 50% in human cells (Fig. S1B). Thus all RNA polymerase mutations except PB1 R190K were shown to be adaptive with respect to increased RNA polymerase activity [74].

**Table 4:** Sites of parallel, positive and nuclear trafficking signal mouse adapted mutations.

gene	sites of parallel evolution/mutations (mutants)^a^	postive^b^	total	nuclear traffic signals
PB2	**482**(3), **701**(27), **740**(9)	6	17	480, 482, 701, 740 (NLS)
PB1	**577**/3(10)	4	8	190 (NLS)
PB1-F2	none	none	none	none
PA	**21**(4), **27**(4), **70**(4), **97**(6), **190**/2(5)	7	13	190 (NLS)
NP	**34**(24), **290**/2(14)	7	13	4 (NLS)
HA	HA1:**162**/2(2), **210**(4), **218**/2(19) HA2:**154**/2(2)+**156**(8)	13	25	na
NA	**110**/2(2)	4	19	na
M1	none	2	3	?
M2	none	1	2	na
NS1	**106**/2(6)	6	10	?
NEP	none	1	5	23 (NES)
total	**17**/27(39)	51	115	8

a format - amino acid positions/number of mutations if >1 (number of mutants among 39 MA clones in parentheses).

b total number of positive and parallel mutations.

NLS, nuclear localization signal.

NES, nuclear export signal.

na, not applicable.

## Discussion

We extended our previous demonstration of parallel and positive evolution in the HA and NS1 genes on IAV adaptation to now include ribonucleocapsid components (PB1, PB2, PA, NP) and NA proteins. In this study, we provide evidence for sites of natural selection in all of these genes (Table 4). We observed that serial high-dose passage of human influenza virus in the mouse lung resulted in the positive selection of mutations. Adaptive mutations clustered in regions of the primary (Fig. 2 and 6) and 3-dimensional structures of viral proteins (Fig. 3–5 and 6–10). We identified 115 mutations distributed among all influenza proteins except PB1-F2, including 27 examples of parallel evolution that primarily involved the polymerase subunits, NP and HA (Table 4). Adaptive mutations were primarily located in regions of interaction with host and in several instances involved sites of viral subunit interaction or oligomerization (NP, NS1, HA, and NA).

### Adaptive mutations affect interaction with host proteins and factors

Mouse adaptive mutations would be expected to affect sites of virus-host interaction, however very few host proteins binding sites have been mapped. As influenza virus replication is nuclear, 8 mutations were found in nuclear trafficking signals [20] of the polymerase subunits, NP, and NEP proteins (Table 4). Mouse adaptive mutations that involved defined nuclear trafficking signal sequences are shown in bold: PB2 736-KRKR(**D740N**)X_11_KRIR-755; PB1 187RKR(**R190K**)VRDNMTKKMVTQRTIGKRKQR211; NP 1MAS(**Q4K**)GTKRSYxxM13; and NEP S23P in NES (11DILLRMSKMQLE(**S23P**)) (Fig. 2 and 6) [20]. The PB2 D701N mutation disrupts a salt bridge with 753R (736-KRKR(X_12_)K**R**IR-755) to result in unfolding of the PB2 NLS structure, (Fig. 3C and 3D) [76] to increase PB2 NLS activity. This is associated with increased binding to mammalian (but not avian) importin 1α and 7α [79] and increased nuclear localization of PB2 and NP proteins [80]. This may have increased nuclear localization activity which in turn would explain the higher polymerase activity seen in the double mutant (PB2 D701N + D740N) (Fig. 11H). The PB2 K482R was also independently selected in the H1N1 MA variant A/FM/1/47-MA where it was shown to enhance virulence (20 fold) and replication in the mouse lung [58].

#### Cap binding domains were primary targets for adaptation

Both the PB2 and PA proteins demonstrated adaptive mutations in their respective cap binding domains, suggesting that increased ability to access host mRNA cap complexes for priming viral transcription is important for overcoming restricted viral replication in a new host. The N-terminal PA cap-binding domain also possesses RNA endonuclease, transcription, replication and protein stability functions that may be affected by mutations in this domain [77].

### Sites of virus-virus protein interaction

Influenza viruses replicate through the action of 11 genes that interact extensively with each other and with host proteins [16]. Adaptive mutations were found to occur extensively in regions that involved contacts with the trimetric RNA-dependent RNA polymerase subunits as well as NP that encapsidates viral RNA in RNP complexes (Fig. 2 and 6). Because the current maps of viral protein interaction are largely incomplete (see [19]) it is possible that the adaptive regions, although concurrent with virus-virus interaction regions, are actually affecting interactions with unknown host factors. We have previously identified adaptive HA mutations that affect subunit interaction to raise the pH of fusion [10]. We have now shown mutations in the NA, NP and NS1 proteins at sites of known viral contact in their crystal structures. These findings indicate that adaptive mutations occur at sites involved in viral protein interactions and oligomerization and may affect properties associated with these interactions (Fig. 2, 5, 7, 8, and 10). The RNA polymerizing subunit, PB1, that binds PB2 and PA, had an adaptive region defined by adjacent amino acid mutations K577Q/E/M and K578T situated in the center of its PB2 interaction domain (Fig. 2). The PB1 Q15H mutation situated adjacent to the amino terminal 14 amino acids in direct contact with the PA subunit binding-cleft could affect this interaction (Fig. 4B). NP protein oligomerization requires contacts mediated by the C-terminal tail linker region that was mutated at multiple sites of NP-NP contact including M426I, A428T, V476A, and D480N (Fig. 5). Future studies will address the roles of these mutations in NP structure and function. The predominance of mutations in RNP components and the increased polymerase activity shown for some of these mutations (Fig. 11 and S1) indicates that increased gene expression is a major driving force in interspecies adaptive evolution. The effect of these adaptive mutations on virus protein interactions and functions remains to be determined.

### NS1 evolution on mouse-adaptation

The NS1 protein is a multifunctional protein that binds RNA and protein factors to antagonize IFN functions and modulate infection [18]. NS1mutations occurred in regions that involve binding sites for dsRNA and factors involved in post transcriptional processing of host mRNA and initiation of protein synthesis as indicated in Fig. 10. NS1mutations between position 98 and 125 with parallel evolution at position 106, involve the eIF4GI/CPSF30 and protein kinase R (PKR) binding sites that enhance viral replication through modification of RNA or protein factor binding to favor viral gene expression (N. E. Forbes and E.G. Brown in preparation). Surprisingly, earlier studies have shown that the F103L and M106I mutations found in the first fatal H5N1human infection in 1997 [81], resulted in a loss of ability of NS1 to bind CPSF30 and inhibit host gene expression [82] as has also been reported for A/PR/8/34 (H1N1) [83]and pandemic 2009 H1N1 viruses [84]. The NS1 F103L and M106I mutations increase replication and virulence in both the A/HK/156/1997(H5N1) and HK/1/68(H3N2) genes [9] indicating that virulence is not dependent on an ability of NS1 to bind CPSF30. Several of the adaptive mutations occurred at or near the sites of NS1subunit contacts in the dimer (F103L, M106I, M106V) and also at sites of contact of the dimer with CPSF30 (F103L, M106I, M106V, V180A) [85], all of which resulted in loss of binding to human CPSF30 (Fig. 10C). Although we expected that these mutations would mediate stronger host protein binding to achieve greater host protein shut-off, reduced CPSF30 binding may decrease inhibition of host gene expression to favor viral replication which requires host mRNA derived capped oligonucleotide primers [86] as well as host proteins [87]. The NS1 mutations were not selected in response to differences in mouse CPSF30 binding site structure because human and mouse CPSF30 (CPSF subunit 4) proteins have identical sequences in the NS1-binding domain (CPSF30-F2/F3 fragment) (Fig. S2). Thus adaptive mutations can lead to the loss of interaction with host proteins. Adaptive mutations therefore included those that either enhanced (PB2 D701N and mammalian importins [79], [80]; HA1 G218^1^W/E and α2,3 sialic acid [10], [88]) or reduced binding to host factors such as CPSF30 (Fig. 10C).

### Surface proteins

The adaptive map of the HA receptor has been previously shown to define clusters of mutations in the HA1 subunit adjacent to the receptor binding pocket in the HA monomer, (included here for reference, Fig. 7A); as well as a domain in the HA2 stalk. Most of the parallel HA mutations (P162^1^S, Q210^1^R, G218^1^W) have been shown to increase the pH of fusion (except T156^2^N) and all of these were associated with increased mouse cell infection and virulence as a function of increased mouse-lung tropism and replication [10]. The G218^2^W mutation increases α2–3 sialic acid (SA) binding, consistent with the fact that α2–3 SA linkages are the only form of SA present in the mouse respiratory tract [89]. In addition to the proximity of both of the HA1 and HA2 adaptive domains to the respective 165^1^ and 154^2^ glycosylation sites (that were both lost due to adaptive mutations) suggests that differences in host mediated glycosylation may also be contributing to HA adaptive evolution (Fig. 7) [90].

The 3D NA adaptive map demonstrated surface mutations that surrounding the active site in the monomer and also subunit contacts in the tetrameric structure that were also proximal to 2 glycosylated sites at aa positions 146 and 200 (Fig. 8); implicating glycosylation and viral (NA-NA) plus host (sialic acid) factor interactions with adaptation. NA mutations also occurred in the C-terminal domain that has been identified in the control of acid stability and avian to mammalian adaptation that may be involved in mouse adaptation [91]–[94].

The M2 ion channel protein had mutations in the ion channel region, including D44N that normally stabilizes the W41 gating amino acid in the closed position[95] (by interacting with both R45 and W41) and the I32T mutation that is adjacent to the important hydrophilic position 31 [96]. Highly pathogenic avian influenza virus has been identified that requires modified M2 protein to prevent premature acid activation of HA fusion in cytoplasmic transport vesicles [97].

### Mutations selected on serial mouse-lung passage were adaptive

In addition to the validation of the adaptive roles of individual mutations selected on serial passage (see introduction), increased RNA polymerase activity was shown for 6 mutations: (PB1 K578T; PB2 D701N, D740N and K482R; and PA Q556R (Fig. 11)). Increased virulence on the basis of body weight loss in the mouse was shown for 7 mutations: PB2 (D701N, D740N K482R), NP (D34N, D290N, and D290E) and PA (Q556R). These observations demonstrate that MA by serial high dose passage is an effective method for identifying adaptive mutations.

### Convergent evolution with human and animal influenza viruses

The PB2 D701N mutation has been demonstrated in MA variants of A/HK/1/68 [59], and H7N7 where it was shown to increase mouse-virulence and polymerase activity [98]. The mutation was also critical for mammalian virulence of a naturally pathogenic avian H5N1 virus [99]. The PB2 D701N mutation has also been observed to be selected in the human respiratory tract on infection with HPAI H5N1 [100] with 10 occurrences of PB2 D701N or D740N mutations (6 and 4 respectively) among 154 human HPAI H5N1 infections currently recorded in GenBank (Table S10). The PB2 D701N mutation may also have been important for adaptation of avian H3N8 viruses to equines because all equine viruses possess PB2 D701N. PB2 D701N has been maintained on adaptation of equine influenza virus to dogs with further evolution to the PB2 D701N + D740N double mutation that may be instrumental in its continued adaptation (Table S10). The PA T97I mutation has also evolved in parallel in MA variants of nonpathogenic avian H5N2 and H7N3 [67], as well as pathogenic H5N1 and H7N1 virus strains [101], [102] where it was shown to be a genetic determinant of increased virulence and polymerase activity. The PB1 K578T mutation (Table 2) evolved in parallel with K578Q that was a determinant of increased virulence and polymerase activity on mouse adaptation of A/equine/London/1416/73(H7N7)[64]. This indicates that MA mutations are selected in multiple species including horses, dogs and humans [100].

### Conclusion

We show that a relatively small number of mutations including those demonstrating parallel evolution mediate mouse adaptation and increased virulence. Many mouse-adapted mutations map to regions of interaction with both host and viral proteins. A group of 18 mutation sites were repeatedly selected and were therefore the most adaptive (Table 4). Thus experimental mouse-adaptation represents a predictable model system for identifying gain-of-function mutations for the identification and characterization of viral protein functions and interactions. Mouse adaptive models are also useful for testing adaptive theories of evolution [4] and supplying reference points for bioinformatics and biochemical studies. Future studies will address the mechanisms of action and gene interactions of adaptive mutations.

## Methods

### Ethics Statement

This study was carried out in compliance with the guidelines of the Canadian Council on Animal Care (CCAC) as outlined in the Care and Use of Experimental Animals, Vol.1, 2nd Edn. (1993), which are recognized as “best-practices” by the International Council for Laboratory Animal Science (ICLAS). The protocol was approved by the University of Ottawa Animal Care Committee (Protocol Number: BMI-85). Animal studies were also performed under the supervision of a veterinarian (DVM) and trained personnel. All efforts were made to minimize suffering and mice were euthanized at humane end-points, if infection resulted in greater than 25% body weight loss plus respiratory distress.

### Cells

Madin-Darby canine kidney cells (MDCK) (Health Canada, Ottawa) were maintained in autoclavable minimum essential medium (MEM) with Earle's salts, and both 293T human embryonic kidney cells (ATCC, Manassas, VA) and mouse B82 fibroblasts (Coriell Institute for Medical Research, Camden, NJ; catalogue number GM00347) were maintained in Dulbecco's MEM (Invitrogen Canada Inc., Burlington). Media were supplemented with L-glutamine (2 mM), Penicillin (100 U/ml), Streptomycin (100 ug/ml) (Invitrogen Canada Inc., Burlington) and fetal bovine serum (FBS) (10%) (Hyclone Laboratories, Utah).

### Mouse serial passage

The prototype clinical isolate A/Hong Kong/1/68 (H3N2) (HK-wt) was obtained from the Laboratory Center for Disease Control, Health Canada, Ottawa that was originally obtained from H.G. Pereira (World Influenza Centre, London). The passage history of HK-wt was: (2 passages in rhesus monkey kidney cells) + (3 passages in chicken allantoic cavity) before 2 plaque purifications (plaque-to-plaque) on MDCK cells before seed and stock preparation in chicken allantoic cavity. The generation of mouse-adapted clones was described previously [59] where 10^5^ pfu HK-wt stock in 50 µL PBS was inoculated intranasally into each of 3, 20 g CD-1 strain mice under halothane anesthesia (3.5% halothane in O_2_), that were housed for 3 days and euthanized by CO_2_ narcosis and surgical removal of lungs. Lungs were pooled in 3 ml PBS and a virus extract prepared by sonication for 2 minutes on ice, before sedimentation of debris by centrifugation at 250 G for 5 minutes. Virus extracts were diluted 10 fold in PBS before inoculating another group of CD-1 mice with 50 µL each with repeated passage for a total of 20 serial passages (all lung extracts were titrated by plaque assay and contained ≥5×10^5^ pfu of virus [24]). Clonal isolates were derived by sequential plaque-to-plaque isolations on MDCK cells from passage 12 (6 clones), and passage 20 (6 clones), and stocks were prepared in MDCK cells or alternatively by a single passage in the allantoic cavity of 10-day-old SPF chicken embryos (Canadian Food Inspection Agency, Ottawa). MA variants were also derived from 9 separate mouse adaption experiments that each involved the serial passage of separate HK-wt stocks produced from HK-wt subclones. The 9 separate HK-wt subclones were obtained by plaque isolation from diluted HK-wt stock and used to produce 9 independent HK-wt stocks that were each used to perform 9 separate mouse passage experiments that employed 21 serial passages each as indicated in Fig. 1. After 21 mouse passages 3 biological clones were derived from each of the 9 “MA populations” by 2 sequential plaque isolations as previously described [10] and shown in Fig. 1. The passage schedule for each of the replicate numbered HK-wt clones (HK(clone #)) involved initial inoculation of individual mice with undiluted stock virus (>10^6^ pfu/mouse) with serial passage of undiluted lung extract into individual mice for a total of 10 passages before 5 serial undiluted passages of 2 days each in groups of 2 mice, followed by 6 serial undiluted passages of 3 days duration in groups of 2 mice. In each of these passages, infected lungs were suspended in 1 ml PBS each to make extracts that were sterilized using 0.22 µM Millipore Millex-GV PVDF (Cork, Ireland) filtration before serial passage that involved infection with 50 µL of lung extract. All MA variants derived from the 9 independent mouse-adapted passage 21 populations clones were designated as HK(clone #) MA (mouse passage #)-(clone #).

### Titration of infectivity by plaque assay

Virus stocks were subjected to serial 10 fold dilution in PBS before application to PBS washed MDCK cells monolayers in 6 well plates as described previously [9]. Average values were calculated for three samples that were each titrated in duplicate plaque assays.

### Calculation of median lethal dose

Median lethal dosage (LD_50_) of IAV variants were determined in groups of CD-1 mice as described previously [10]. Groups of 5 female (19–21 g) CD-1 mice were infected under halothane anesthesia (2.5% in O_2_) with undiluted stock virus and serial 10 fold dilutions made in PBS. Mice were monitored for survival and weight loss over a 2 week period or until body weight increased. Mice that lost >25% body weight and were in respiratory distress where considered to have reached “humane endpoint” as required by our animal care protocol and were euthanized by CO_2_ narcosis. The median lethal dose (LD_50_) was determined using the Karber–Spearman method, using the formula (negative log_10_ of LD_50_)  =  (negative log_10_ of highest dose) – (–((sum of percent mortality at each dose/100) – 0.5)) ×log_10_ of dilution steps)[103]. The standard deviation of LD_50_ values calculated from n = 3 independent assays using groups of 5 CD-1 mice, has been determined to be 10^0.3^ pfu [57].

### Statistical analysis

Sample values were calculated as averages ± standard deviation for sample size ≥3 with statistical significance at the P ≤ 0.05 level determined using the paired or single sample, 2-tailed student's t-test where indicated, using the Microsoft Office Excel® 2007 or Graphpad Prism® v3.02 programs.

### Population size required to contain all possible single nucleotide polymorphisms (SNP)

There are 13,629 nucleotides per HK-wt genome with 3 possible substitutions at each site to yield 13,629×3 = 40,887 SNP's. The observed mutation rate of 1.5×10^−5^ per nucleotide per replication cycle [104], or ((1.5×10^−5^) ×13,629 ntds)  = 0.2 mutations per genome (Table 1)), therefore the total number of 40,887 SNP's is predicted to be present in a population of 2.0×10^5^ infectious virions (40,887 SNP/(0.2 mutations per genome). Virus populations of 2.0×10^5^ infectious viruses are expected to possess all possible single amino acid substitution mutations (multiple combinations of 2 or more SNP's are too rare to be relevant to molecular evolution [44]).

### Calculation of the random probability of isolation of multiple variants with identical mutations

Positive selection results in the increased prevalence of mutation due to enhanced fitness versus random occurrence of the same mutations. In analysis of positive selection the null hypothesis is that the mutants occur at a frequency predicted by random probability or chance. Given that prototype A/HK/1/68 virus has a genome of 13,629 nucleotides and each position can be substituted with 3 alternative nucleotides, there are 3 times genome length (13,629×3) or 40,887 possible SNP variants. Thus any SNP mutant has a random probability of occurrence of 1/40,887 and thus the probability that multiple strains of influenza (n) will have the same mutation (SNP) is the product of the individual probabilities times the number of samples tested (N) to get (N/40,887)^n^ (see reference [44])that is P≤2×10^−8^ for 2 or more identical mutations in a population of 6 viruses which is much less than the significance limit of p = 0.05 and thus causes rejection of the null hypothesis in favor of positive selection. Similarly the probability of ≥2 identical mutation among multiple populations composed of 36 viruses (the largest N in this manuscript) has P =  (36/40,887) ≥^2^ or P≤2×10^−5^ again leading to rejection of the null hypothesis to support positive selection. Thus the selection of ≥2 mutants with the same mutation in the same or different populations is strong evidence of positive selection indicating that the mutation was positively selected and therefore adaptive.

### Reverse genetics

HK-wt and mutants were produced using the 8 plasmid recombineering approach with pLLB plasmids [105] as describe previously [74].

### Sequencing

Viral RNA was extracted from 140 µL of stock allantoic fluid from each virus using the QIAamp Viral RNA Mini Kit (Qiagen, Mississauga, Ontario) and full length influenza genomic segments were amplified [106], sequenced, and assembled as previously described [107], [108]. All Genbank accession numbers are listed in Table S9.

### RNA Polymerase assays in human and mouse cells

To compare the activities of viral RNP complexes in human and mouse cells, a Promega Dual-Glo Luciferase Assay System (Promega) was used [98], [109]. A luciferase reporter minigenome polymerase assay was constructed that possessed the firefly luciferase gene driven by the human RNA POL I promoter (phPOLI-NP-LUC) and mouse RNA POL I terminator to generate a luciferase negative sense transcript flanked by the influenza NP gene noncoding regions.

The phPOL1-NP-LUC plasmid was constructed by insertion of the firefly luciferase gene and NP non-coding regions that were amplified by PCR using pGL3 Basic (Promega, Fisher Scientific, Nepean, Ont.) as template and the following primers: Fw: 5′TATTCGTCTCAGGGAGCAAAAGCAGGGTAGATAATCACTCACTGAGTGACATCAAAATCATGGAAGACGCCAAAAACATA-3′ Bw: 5′ATATCGTCTCGTATTAGTAGAAACAAGGGTATTTTTCTTTACACGGCGATCTTTCCG-3′. The PCR product was digested by BsmBI and cloned into BsmBI digested pHH21, resulting in plasmid phPOL1-NP-LUC.

The pmPOL1-NP-LUC plasmid (mouse Polymerase I promoter-Luc construct) was constructed by PCR amplification of the firefly luciferase gene flanked by NP non-coding region using the pGL3 Basic plasmid (Promega) as template and the following primers:Fw:5′ATATCGTCTCAGGGAGCAAAAGCAGGGTAGATAATCACTCACTGAGTGACATCAAAATCATGGAAGACGCCAAAAACAT-3′, Bw: 5′-TATTCGTCTCAAGGTAGTAGAAACAAGGGTATTTTTCTTTACACGGCGATCTTTCCGC-3′. The PCR product was digested by BsmBI and cloned into BsmBI digested pHL1261 [110], resulting in plasmid pmPOL1-NP-LUC. Plasmids were sequenced to ensure there were no unwanted mutations.

To perform the luciferase assays, 96 well plates of human 293T or mouse B82 cells were respectively transfected with 0.06 µg of the reporter plasmids, phPOLI-NP-LUC or pmPOLI-NP-LUC, in combination with 0.06 µg of each of the four pLLB-plasmids encoding the HK-wt or mutant forms of PB2, PB1, PA, NP[105] plus 0.06 µg of the internal control renilla luciferase expression plasmid PRL-SV40 (Promega), using 0.5 µL of lipofectin 2000 in 100 µL of Opti-MEM (Invitrogen, Burlington, Ontario). At 48 h post-transfection, luminescence was measured using the Promega Dual-Glo Luciferase Assay System and a Glomax Multi Detection System, Model 9301-010 (Fisher Scientific, Nepean, Ont.) according to the manufacturer's instructions. Relative luciferase activities were calculated as the average + standard deviation of the ratios of firefly and renilla luciferase luminescence for three independent experiments of 3 replicates each.

### Genomic mapping of mutations

Nucleotide and amino acid sequences were aligned to identify mutations using BioEdit version 7.0.5.3 and Genedoc Multiple sequence alignment Editor and Shading Utility version 2.7.000 software.

### Linear mapping of mutations

For each influenza protein, adaptive mutations were positioned according to their amino acid sequence location onto linear primary structural maps that indicated known site of function or interaction with other viral and host factors. Linear maps were made with CorelDRAW 10 v10.410 software. The linear maps of PB2, PB1, PA, and NP were derived from the previous maps of Boulo et al [20] as modified by Naffakh et al., 2008 [19] with pertinent references therein. The HA primary structure element maps of the active site were derived from the reviews of Skehel and Wiley [111] and Stevens et al [112]. The NA domains were derived from the crystal structure [113] and the review of Nayak and Jabbar [114].The M1 protein map was composed from data in [115]–[119]. The M2 ion channel was mapped from data of Lamb et al, [120]. The primary structural map of NS1 was modified from the map of Hale et al., [18] and references therein. The location of the NEP nuclear export signal was from [121].

### Three dimensional structural maps

Structural maps were generated using the PDB ProteinWorkshop version 3.7 [122] with protein shown in ribbon diagram with numbered mutations in space filling models. Maps used the following structural files: PB2-(aa 535–742) PDB ID 3CW4, PB2-C-terminus (aa 688–756) PDB ID 2GMO, PB2-C-terminus (aa 686–757) human importin α5 co-crystal PDB ID 2JDQ; PB2-(aa 320–483)-7methy guanosine cap co-crystal, PDB ID 2VQZ; PA (aa 257–716) bound to amino terminus of PB1 (aa 1–16) co-crystal PDB ID 3CM8; PA amino-terminal domain (aa 1–209) PDB ID 2W69; H3 HA (HA1 aa1–328, HA2 aa 1–175) PDB ID 1HDG; H3 low pH form (aa34–178) PDB ID 1QU1; NP trimer (aa 8–498) PDB ID 2IGH; NA2 PDB ID 1NN2; M2-(aa 23–60) PDB ID 2KIH; NS1-(aa 1–215) PDB ID 3F5T; NS1-(aa 85–203)-CPSF30-F2F3-(aa 56–118) co-crystal PDB ID 2RHK; and NS2-(aa 63–116) PDB ID 1PD3.

### Protein Gel Electrophoresis and Western blot

Samples were fractionated by SDS PAGE using 12.5% acrylamide gels as described previously [57]. Western blots employed rabbit antiserum raised against purified recombinant A/HK/1/68 NS1 protein or anti-FLAG M1 mouse monoclonal antibody (Sigma Chemical, Burlington) and were performed as described previously [123] but were detected with HRP conjugated goat-anti-rabbit or goat-anti-mouse (Sigma Chemical, Burlington) respectively, and SuperSignal West Pico chemiluminescent substrate (Pierce). Quantification employed densitometry using the UN-SCAN-IT Gel version 6.1 software (Silk Scientific Corp).

### NS1 CPSF30 binding assay

Recombinant NS1 proteins with amino terminal 6xHis tags were synthesized as described previously [123] in BL21 pLysS *E. coli* using pET17b plasmids for 16 h at 21°C with 10 µM of IPTG except that the soluble fraction was employed for purification and was dialyzed against PBS. Purified NS1 protein was quantified using the Bio-Rad Protein Assay and standardized by comparative western blot. Plasmids were constructed by insertion of the NS1 genes of HK-wt and each mutant produced by PCR mutagenesis into pET17b after PCR amplification using pfu Turbo polymerase (Stratagene, La Jolla, CA). CPSF30 or the CPSF30-F2F3 fragment was expressed in 1.5×10^7^ 293T cells transfected with 30 µg of pCAGGS-CPSF30-Flag or pCAGGS-CPSF30-F2F3-Flag plasmid (obtained from L. Martinez-Sobrido, Mt. Sinai school of Medicine) in 112 µl of Lipofectamine 2000 transfection reagent (Invitrogen, Burlington, Ont.) for 24 hrs before lysis with 100 mM Tris, 250 mM NaCl, 0.5% NP-40, and 0.5% DOC, pH 8.5. Pull down experiments employed the lysate from 5×10^5^ 293 T cells, 1 ug of anti-FLAG M1 monoclonal antibody (Sigma-Aldrich, Canada), defined amounts of NS1 protein, and 20 µl of protein G Dyna-beads (Invitrogen, Burlington, Ont,) in a 0.25 ml volume with rotation for 2 hr at room temperature. Beads were washed three times in lysis buffer for 10 minutes before western blotting. Control pull-down assays used the lysate from 5×10^5^ 293 T cells transfected with empty vector that did not result in NS1 pulldown indicating a lack of nonspecific binding to anti-FLAG M1 monoclonal antibody. Similar results were obtained for both CPSF30 or the CPSF30-F2F3 fragment pull-downs and therefore only the CPSF-F2F3 fragment data were shown. Bound NS1 proteins were detected by western blotting with rabbit anti-NS1 antibody. Anti-FLAG M1 monoclonal antibody was monitored after pull-down by Coomassie Brilliant Blue staining of samples separated on 12.5% SDS-PAGE gels.

## Supporting Information

Table S1 **Mutations found in previous studies of mouse adaptation of IAV.**
(DOC)

Table S2 **Number of mutations selected in each of 39 mouse adapted variants from 10 replicate mouse adaptation experiments.**
(DOC)

Table S3 **Amino acid changes in the PB2 protein of parental HK clones and their corresponding mouse adapted clones derived after 21 serial passages in the mouse lung.**
(DOC)

Table S4 **Amino acid changes in the PB1 and PA proteins of HK-wt clones and their corresponding mouse adapted variants derived after 21 serial passages in the mouse lung.**
(DOC)

Table S5 **Amino acid changes in the NP protein of parental HK clones and their corresponding mouse adapted clones derived after 21 serial passages in the mouse lung.**
(DOC)

Table S6 **Amino acid changes in the HA protein of parental HK clones and their corresponding mouse adapted clones derived after 21 serial passages in the mouse lung.**
(DOC)

Table S7 **Amino acid changes in the NA protein of parental HK clones and their corresponding mouse adapted clones derived after 21 serial passages in the mouse lung.**
(DOC)

Table S8 **Amino acid changes in the M1, M2, NS1, and NEP proteins of parental HK clones and mouse adapted clones derived after 21 serial passages in the mouse lung.**
(DOC)

Table S9 **List of Genbank accession numbers for nucleotide gene sequences of HK parental and mouse adapted variant clones for each genome segment with encoded proteins and nucleotide sequence length indicated.**
(DOC)

Table S10 **List of PB2 gene Genbank accession numbers for human H5N1 and canine H3N8 isolates that possess PB2 D701N and/or PB2 D740N mutations.**
(DOC)

Figure S1 **RNA polymerase activity effects of PB1, PB2, PA and NP mutations in mouse and human cells.** Polymerase activity is shown for B82 mouse cells (A) and human 293T cells (B). Mouse adaptive mutations at the indicated positions are masked in gray and HK-wt is shown without mask in the table aligned with bars of activity. Influenza luciferase assays employed luciferase minigenomes expressed via a human or mouse POL1 polymerase in mouse B82 and human 293T cells respectively. Samples (i) has the HKMA12A and 12D combination of mutations; (j) has HKMA12E; and (m) has the HKMA20B, C, and D combination. Values were standardized relative to HK wt luciferase activity as 100% and are shown as averages for n = 3 experiments ± SD. Asterisks indicates significant difference from HK-wt polymerase activity for each cell type (* and ** indicate P≤0.05 and P≤0.01 by t-test respectively).
(TIF)

Figure S2 **Blast alignment of mouse CPSF30 with human CPSF30.** Amino acid sequence of mouse CPSF30 (query) is aligned above human CPSF30 sequence (Sbjct) with the consensus sequence indicated between each sequence. The F2F3 binding fragment is indicated in yellow mask showing identical amino acid sequence between human and mouse.
(PDF)
