# Supplementary material for: Genomic and Protein Structural Maps of Adaptive Evolution of Human Influenza A Virus to Increased Virulence in the Mouse
Source: PLoS One. 2011 Jun 30;6(6):e21740. doi: 10.1371/journal.pone.0021740 (PMC3128085; doi:10.1371/journal.pone.0021740)
Supplement: Table S1 — Mutations found in previous studies of mouse adaptation of IAV. (DOC) [file pone.0021740.s001.doc]

**Table S1. Mutations found in previous studies of mouse adaptation of IAV.**

a, superscripts denote HA subunits 1 and 2

ns, not specified

mutations in red have been shown to increase virulence

data from references [1–15]

Reference List

1. Brown EG, Bailly JE (1999) Genetic analysis of mouse-adapted influenza A virus identifies roles for the NA, PB1, and PB2 genes in virulence. Virus Res 61: 63-76.

2. Brown EG, Liu H, Kit LC, Baird S, Nesrallah M (2001) Pattern of mutation in the genome of influenza A virus on adaptation to increased virulence in the mouse lung: identification of functional themes. Proc Natl Acad Sci U S A 98: 6883-6888.

3. Gabriel G, Dauber B, Wolff T, Planz O, Klenk HD, Stech J (2005) The viral polymerase mediates adaptation of an avian influenza virus to a mammalian host. Proc Natl Acad Sci U S A %20;102: 18590-18595.

4. Ilyushina NA, Khalenkov AM, Seiler JP, Forrest HL, Bovin NV, Marjuki H, Barman S, Webster RG, Webby RJ (2010) Adaptation of pandemic H1N1 influenza viruses in mice. J Virol 84: 8607-8616. JVI.00159-10 [pii];10.1128/JVI.00159-10 [doi].

5. Li J, Ishaq M, Prudence M, Xi X, Hu T, Liu Q, Guo D (2009) Single mutation at the amino acid position 627 of PB2 that leads to increased virulence of an H5N1 avian influenza virus during adaptation in mice can be compensated by multiple mutations at other sites of PB2. Virus Res 144: 123-129.

6. Narasaraju T, Sim MK, Ng HH, Phoon MC, Shanker N, Lal SK, Chow VT (2009) Adaptation of human influenza H3N2 virus in a mouse pneumonitis model: insights into viral virulence, tissue tropism and host pathogenesis. Microbes Infect 11: 2-11.

7. Ping J, Dankar SK, Forbes NE, Keleta L, Zhou Y, Tyler S, Brown EG (2010) PB2 and HA Mutations are Major Determinants of Host Range and Virulence in Mouse-Adapted Influenza A Virus. J Virol . JVI.01187-10 [pii];10.1128/JVI.01187-10 [doi].

8. Rolling T, Koerner I, Zimmermann P, Holz K, Haller O, Staeheli P, Kochs G (2009) Adaptive mutations resulting in enhanced polymerase activity contribute to high virulence of influenza A virus in mice. J Virol 83: 6673-6680.

9. Shinya K, Watanabe S, Ito T, Kasai N, Kawaoka Y (2007) Adaptation of an H7N7 equine influenza A virus in mice. J Gen Virol 88: 547-553.

10. Smee DF, Wandersee MK, Checketts MB, O'Keefe BR, Saucedo C, Boyd MR, Mishin VP, Gubareva LV (2007) Influenza A (H1N1) virus resistance to cyanovirin-N arises naturally during adaptation to mice and by passage in cell culture in the presence of the inhibitor. Antivir Chem Chemother 18: 317-327.

11. Ward AC (1995) Specific changes in the M1 protein during adaptation of influenza virus to mouse. Arch Virol 140: 383-389.

12. Ye J, Sorrell EM, Cai Y, Shao H, Xu K, Pena L, Hickman D, Song H, Angel M, Medina RA, Manicassamy B, Garcia-Sastre A, Perez DR (2010) Variations in the hemagglutinin of the 2009 H1N1 pandemic virus: potential for strains with altered virulence phenotype? PLoS Pathog 6: e1001145. 10.1371/journal.ppat.1001145 [doi].

13. Zhou B, Li Y, Halpin R, Hine E, Spiro DJ, Wentworth DE (2011) PB2 residue 158 is a pathogenic determinant of pandemic H1N1 and H5 influenza a viruses in mice. J Virol 85: 357-365. JVI.01694-10 [pii];10.1128/JVI.01694-10 [doi].

14. Dankar SK, Wang S, Ping J, Forbes NE, Keleta L, Li Y, Brown EG (2011) Influenza A virus NS1 gene mutations F103L and M106I increase replication and virulence. Virol J 8: 13. 1743-422X-8-13 [pii];10.1186/1743-422X-8-13 [doi].

15. Keleta L, Ibricevic A, Bovin NV, Brody SL, Brown EG (2008) Experimental evolution of human influenza virus H3 hemagglutinin in the mouse lung identifies adaptive regions in HA1 and HA2. J Virol 82: 11599-11608.
