# Supplementary material for: Genomic and Protein Structural Maps of Adaptive Evolution of Human Influenza A Virus to Increased Virulence in the Mouse
Source: PLoS One. 2011 Jun 30;6(6):e21740. doi: 10.1371/journal.pone.0021740 (PMC3128085; doi:10.1371/journal.pone.0021740)
Supplement: Table S2 — Number of mutations selected in each of 39 mouse adapted variants from 10 replicate mouse adaptation experiments. (DOC) [file pone.0021740.s002.doc]

**Table S2.** **Number of mutations selected in each of 39 mouse adapted variants from 10 replicate mouse adaptation experiments.**

Clones from specific populations are indicated as MA-12, MA-20, or

MA21 population#-clone #.

nd, not done

a, mouse median lethal dose values for HKMA12 and MA20 strains from [1]

b, geometric mean of log_10_ values

Reference List

1. Brown EG, Liu H, Kit LC, Baird S, Nesrallah M (2001) Pattern of mutation in the genome of influenza A virus on adaptation to increased virulence in the mouse lung: identification of functional themes. Proc Natl Acad Sci U S A 98: 6883-6888.
