# Supplementary material for: Genomic and Protein Structural Maps of Adaptive Evolution of Human Influenza A Virus to Increased Virulence in the Mouse
Source: PLoS One. 2011 Jun 30;6(6):e21740. doi: 10.1371/journal.pone.0021740 (PMC3128085; doi:10.1371/journal.pone.0021740)
Supplement: Table S5 — Amino acid changes in the NP protein of parental HK clones and their corresponding mouse adapted clones derived after 21 serial passages in the mouse lung. (DOC) [file pone.0021740.s005.doc]

**Table S5. Amino acid changes in the NP protein of parental HK clones and their corresponding mouse adapted clones derived after 21 serial passages in the mouse lung.**

dots indicate identity to HK-wt aa.

pos. sel., indicates evidence of positive selection, indicated in red.

parallel, mutations that were selected independently in multiple populations in red.

y, indicates yes.

nd, not detected.

na, not applicable.
