# Supplementary material for: Genomic and Protein Structural Maps of Adaptive Evolution of Human Influenza A Virus to Increased Virulence in the Mouse
Source: PLoS One. 2011 Jun 30;6(6):e21740. doi: 10.1371/journal.pone.0021740 (PMC3128085; doi:10.1371/journal.pone.0021740)
Supplement: Table S6 — Amino acid changes in the HA protein of parental HK clones and their corresponding mouse adapted clones derived after 21 serial passages in the mouse lung. (DOC) [file pone.0021740.s006.doc]

**Table S6.** **Amino acid changes in the HA protein of parental HK clones and their corresponding mouse adapted clones derived after 21 serial passages in the mouse lung.**

dots indicate identity to HK-wt aa.

pos. sel., indicates evidence of positive selection indicated in red.

parallel, mutations that were selected independently in multiple populations in red.

a, this mutation was parallel to that of MA12B (Table 2).

y, indicates yes.

nd, not detected. na, not applicable.

These HA genes had been independently sequenced previously [1].

Reference List

1. Keleta L, Ibricevic A, Bovin NV, Brody SL, Brown EG (2008) Experimental evolution of human influenza virus H3 hemagglutinin in the mouse lung identifies adaptive regions in HA1 and HA2. J Virol 82: 11599-11608.
