# Supplementary material for: Genomic and Protein Structural Maps of Adaptive Evolution of Human Influenza A Virus to Increased Virulence in the Mouse
Source: PLoS One. 2011 Jun 30;6(6):e21740. doi: 10.1371/journal.pone.0021740 (PMC3128085; doi:10.1371/journal.pone.0021740)
Supplement: Table S8 — Amino acid changes in the M1, M2, NS1, and NEP proteins of parental HK clones and mouse adapted clones derived after 21 serial passages in the mouse lung. (DOC) [file pone.0021740.s008.doc]

**Table S8.** **Amino acid changes in the M1, M2, NS1, and NEP proteins of parental HK clones and mouse adapted clones derived after 21 serial passages in the mouse lung.**

dots indicate identity to HK-wt aa.

pos. sel., indicates evidence of positive selection indicated in red.

parallel, mutations that were selected independently in multiple populations in red.

y, indicates yes.

nd, not detected. na, not applicable.

19 NS1 genes were independently sequenced and published in a previous study[1].

Reference List

1. Dankar SK, Wang S, Ping J, Forbes NE, Keleta L, Li Y, Brown EG (2011) Influenza A virus NS1 gene mutations F103L and M106I increase replication and virulence. Virol J 8: 13. 1743-422X-8-13 [pii];10.1186/1743-422X-8-13 [doi].
