# Supplementary material for: Genomic and Protein Structural Maps of Adaptive Evolution of Human Influenza A Virus to Increased Virulence in the Mouse
Source: PLoS One. 2011 Jun 30;6(6):e21740. doi: 10.1371/journal.pone.0021740 (PMC3128085; doi:10.1371/journal.pone.0021740)
Supplement: Table S9 — List of Genbank accession numbers for nucleotide gene sequences of HK parental and mouse adapted variant clones for each genome segment with encoded proteins and nucleotide sequence length indicated. (DOC) [file pone.0021740.s009.doc]

Table S9. List of Genbank accession numbers for nucleotide gene sequences of HK parental and mouse adapted variant clones for each genome segment with encoded proteins and nucleotide sequence length indicated.

| Virus clone name | segment (protein) | nucleotides | Accession No. |
| --- | --- | --- | --- |
| A/Hong Kong/1/1968(H3N2) | 1 (PB2) | 2284 | CY044268 |
| A/Hong Kong/1-1/1968(H3N2) | 1 (PB2) | 2296 | CY033008 |
| A/Hong Kong/1-2/1968(H3N2) | 1 (PB2) | 2297 | CY033016 |
| A/Hong Kong/1-4/1968(H3N2) | 1 (PB2) | 2296 | CY033024 |
| A/Hong Kong/1-5/1968(H3N2) | 1 (PB2) | 2301 | CY033032 |
| A/Hong Kong/1-6/1968(H3N2) | 1 (PB2) | 2296 | CY033040 |
| A/Hong Kong/1-8/1968(H3N2) | 1 (PB2) | 2301 | CY034003 |
| A/Hong Kong/1-9/1968(H3N2) | 1 (PB2) | 2301 | CY033056 |
| A/Hong Kong/1-11/1968(H3N2) | 1 (PB2) | 2297 | CY034011 |
| A/Hong Kong/1-12/1968(H3N2) | 1 (PB2) | 2295 | CY033072 |
| A/Hong Kong/1-1-MA-12/1968(H3N2) | 1 (PB2) | 2296 | CY033512 |
| A/Hong Kong/1-1-MA-12A/1968(H3N2) | 1 (PB2) | 2282 | CY034949 |
| A/Hong Kong/1-1-MA-12B/1968(H3N2) | 1 (PB2) | 2292 | CY034957 |
| A/Hong Kong/1-1-MA-12C/1968(H3N2) | 1 (PB2) | 2295 | CY034965 |
| A/Hong Kong/1-1-MA-12D/1968(H3N2) | 1 (PB2) | 2297 | CY033995 |
| A/Hong Kong/1-1-MA-12E/1968(H3N2) | 1 (PB2) | 2302 | CY034973 |
| A/Hong Kong/1-1-MA-20/1968(H3N2) | 1 (PB2) | 2296 | CY044276 |
| A/Hong Kong/1-1-MA-20A/1968(H3N2) | 1 (PB2) | 2301 | CY032984 |
| A/Hong Kong/1-1-MA-20B/1968(H3N2) | 1 (PB2) | 2301 | CY032992 |
| A/Hong Kong/1-1-MA-20C/1968(H3N2) | 1 (PB2) | 2303 | CY045723 |
| A/Hong Kong/1-1-MA-20D/1968(H3N2) | 1 (PB2) | 2301 | CY034981 |
| A/Hong Kong/1-1-MA-20E/1968(H3N2) | 1 (PB2) | 2299 | CY033000 |
| A/Hong Kong/1-1-MA21-1/1968(H3N2) | 1 (PB2) | 2297 | CY033080 |
| A/Hong Kong/1-1-MA21-2/1968(H3N2) | 1 (PB2) | 2296 | CY033088 |
| A/Hong Kong/1-1-MA21-3/1968(H3N2) | 1 (PB2) | 2296 | CY033096 |
| A/Hong Kong/1-2-MA21-1/1968(H3N2) | 1 (PB2) | 2297 | CY033104 |
| A/Hong Kong/1-2-MA21-2/1968(H3N2) | 1 (PB2) | 2296 | CY033112 |
| A/Hong Kong/1-2-MA21-3/1968(H3N2) | 1 (PB2) | 2313 | HM641140 |
| A/Hong Kong/1-4-MA21-1/1968(H3N2) | 1 (PB2) | 2313 | HM641139 |
| A/Hong Kong/1-4-MA21-2/1968(H3N2) | 1 (PB2) | 2297 | CY033120 |
| A/Hong Kong/1-4-MA21-3/1968(H3N2) | 1 (PB2) | 2294 | CY033128 |
| A/Hong Kong/1-5-MA21-1/1968(H3N2) | 1 (PB2) | 2313 | HM641138 |
| A/Hong Kong/1-5-MA21-2/1968(H3N2) | 1 (PB2) | 2301 | CY033136 |
| A/Hong Kong/1-5-MA21-3/1968(H3N2) | 1 (PB2) | 2295 | CY044284 |
| A/Hong Kong/1-6-MA21-1/1968(H3N2) | 1 (PB2) | 2286 | CY044292 |
| A/Hong Kong/1-6-MA21-2/1968(H3N2) | 1 (PB2) | 2296 | CY034019 |
| A/Hong Kong/1-6-MA21-3/1968(H3N2) | 1 (PB2) | 2280 | CY045739 |
| A/Hong Kong/1-8-MA21-1/1968(H3N2) | 1 (PB2) | 2301 | CY034027 |
| A/Hong Kong/1-8-MA21-2/1968(H3N2) | 1 (PB2) | 2296 | CY033144 |
| A/Hong Kong/1-8-MA21-3/1968(H3N2) | 1 (PB2) | 2294 | CY034035 |
| A/Hong Kong/1-9-MA21-1/1968(H3N2) | 1 (PB2) | 2291 | CY033520 |
| A/Hong Kong/1-9-MA21-2/1968(H3N2) | 1 (PB2) | 2298 | CY033528 |
| A/Hong Kong/1-9-MA21-3/1968(H3N2) | 1 (PB2) | 2301 | CY033536 |
| A/Hong Kong/1-11-MA21-1/1968(H3N2) | 1 (PB2) | 2291 | CY033544 |
| A/Hong Kong/1-11-MA21-2/1968(H3N2) | 1 (PB2) | 2296 | CY033552 |
| A/Hong Kong/1-11-MA21-3/1968(H3N2) | 1 (PB2) | 2297 | CY033560 |
| A/Hong Kong/1-12-MA21-1/1968(H3N2) | 1 (PB2) | 2301 | CY033568 |
| A/Hong Kong/1-12-MA21-2/1968(H3N2) | 1 (PB2) | 2295 | CY033576 |
| A/Hong Kong/1-12-MA21-3/1968(H3N2) | 1 (PB2) | 2289 | CY034043 |
|  |  |  |  |
| Virus clone name | segment (protein) | nucleotides | Accession No. |
| A/Hong Kong/1/1968(H3N2) | 2 (PB1) | 2285 | CY044267 |
| A/Hong Kong/1-1/1968(H3N2) | 2 (PB1) | 2300 | CY033007 |
| A/Hong Kong/1-2/1968(H3N2) | 2 (PB1) | 2299 | CY033015 |
| A/Hong Kong/1-4/1968(H3N2) | 2 (PB1) | 2299 | CY033023 |
| A/Hong Kong/1-5/1968(H3N2) | 2 (PB1) | 2303 | CY033031 |
| A/Hong Kong/1-6/1968(H3N2) | 2 (PB1) | 2299 | CY033039 |
| A/Hong Kong/1-8/1968(H3N2) | 2 (PB1) | 2299 | CY034002 |
| A/Hong Kong/1-9/1968(H3N2) | 2 (PB1) | 2297 | CY033055 |
| A/Hong Kong/1-11/1968(H3N2) | 2 (PB1) | 2285 | CY034010 |
| A/Hong Kong/1-12/1968(H3N2) | 2 (PB1) | 2284 | CY033071 |
| A/Hong Kong/1-1-MA-12/1968(H3N2) | 2 (PB1) | 2282 | CY033511 |
| A/Hong Kong/1-1-MA-12A/1968(H3N2) | 2 (PB1) | 2289 | CY034948 |
| A/Hong Kong/1-1-MA-12B/1968(H3N2) | 2 (PB1) | 2284 | CY034956 |
| A/Hong Kong/1-1-MA-12C/1968(H3N2) | 2 (PB1) | 2284 | CY034964 |
| A/Hong Kong/1-1-MA-12D/1968(H3N2) | 2 (PB1) | 2284 | CY033994 |
| A/Hong Kong/1-1-MA-12E/1968(H3N2) | 2 (PB1) | 2285 | CY034972 |
| A/Hong Kong/1-1-MA-20/1968(H3N2) | 2 (PB1) | 2283 | CY044275 |
| A/Hong Kong/1-1-MA-20A/1968(H3N2) | 2 (PB1) | 2290 | CY032983 |
| A/Hong Kong/1-1-MA-20B/1968(H3N2) | 2 (PB1) | 2296 | CY032991 |
| A/Hong Kong/1-1-MA-20C/1968(H3N2) | 2 (PB1) | 2293 | CY045722 |
| A/Hong Kong/1-1-MA-20D/1968(H3N2) | 2 (PB1) | 2290 | CY034980 |
| A/Hong Kong/1-1-MA-20E/1968(H3N2) | 2 (PB1) | 2284 | CY032999 |
| A/Hong Kong/1-1-MA21-1/1968(H3N2) | 2 (PB1) | 2278 | CY033079 |
| A/Hong Kong/1-1-MA21-2/1968(H3N2) | 2 (PB1) | 2299 | CY033087 |
| A/Hong Kong/1-1-MA21-3/1968(H3N2) | 2 (PB1) | 2297 | CY033095 |
| A/Hong Kong/1-2-MA21-1/1968(H3N2) | 2 (PB1) | 2301 | CY033103 |
| A/Hong Kong/1-2-MA21-2/1968(H3N2) | 2 (PB1) | 2300 | CY033111 |
| A/Hong Kong/1-2-MA21-3/1968(H3N2) | 2 (PB1) | 2341 | HM641151 |
| A/Hong Kong/1-4-MA21-1/1968(H3N2) | 2 (PB1) | 2341 | HM641150 |
| A/Hong Kong/1-4-MA21-2/1968(H3N2) | 2 (PB1) | 2284 | CY033119 |
| A/Hong Kong/1-4-MA21-3/1968(H3N2) | 2 (PB1) | 2297 | CY033127 |
| A/Hong Kong/1-5-MA21-1/1968(H3N2) | 2 (PB1) | 2341 | HM641149 |
| A/Hong Kong/1-5-MA21-2/1968(H3N2) | 2 (PB1) | 2284 | CY033135 |
| A/Hong Kong/1-5-MA21-3/1968(H3N2) | 2 (PB1) | 2284 | CY044283 |
| A/Hong Kong/1-6-MA21-1/1968(H3N2) | 2 (PB1) | 2289 | CY044291 |
| A/Hong Kong/1-6-MA21-2/1968(H3N2) | 2 (PB1) | 2297 | CY034018 |
| A/Hong Kong/1-6-MA21-3/1968(H3N2) | 2 (PB1) | 2286 | CY045738 |
| A/Hong Kong/1-8-MA21-1/1968(H3N2) | 2 (PB1) | 2300 | CY034026 |
| A/Hong Kong/1-8-MA21-2/1968(H3N2) | 2 (PB1) | 2303 | CY033143 |
| A/Hong Kong/1-8-MA21-3/1968(H3N2) | 2 (PB1) | 2297 | CY034034 |
| A/Hong Kong/1-9-MA21-1/1968(H3N2) | 2 (PB1) | 2303 | CY033519 |
| A/Hong Kong/1-9-MA21-2/1968(H3N2) | 2 (PB1) | 2305 | CY033527 |
| A/Hong Kong/1-9-MA21-3/1968(H3N2) | 2 (PB1) | 2299 | CY033535 |
| A/Hong Kong/1-11-MA21-1/1968(H3N2) | 2 (PB1) | 2302 | CY033543 |
| A/Hong Kong/1-11-MA21-2/1968(H3N2) | 2 (PB1) | 2299 | CY033551 |
| A/Hong Kong/1-11-MA21-3/1968(H3N2) | 2 (PB1) | 2304 | CY033559 |
| A/Hong Kong/1-12-MA21-1/1968(H3N2) | 2 (PB1) | 2299 | CY033567 |
| A/Hong Kong/1-12-MA21-2/1968(H3N2) | 2 (PB1) | 2301 | CY033575 |
| A/Hong Kong/1-12-MA21-3/1968(H3N2) | 2 (PB1) | 2283 | CY034042 |
|  |  |  |  |
| Virus clone name | segment (protein) | nucleotides | Accession No. |
| A/Hong Kong/1/1968(H3N2) | 3 (PA) | 2183 | CY044266 |
| A/Hong Kong/1-1/1968(H3N2) | 3 (PA) | 2189 | CY033006 |
| A/Hong Kong/1-2/1968(H3N2) | 3 (PA) | 2189 | CY033014 |
| A/Hong Kong/1-4/1968(H3N2) | 3 (PA) | 2189 | CY033022 |
| A/Hong Kong/1-5/1968(H3N2) | 3 (PA) | 2191 | CY033030 |
| A/Hong Kong/1-6/1968(H3N2) | 3 (PA) | 2189 | CY033038 |
| A/Hong Kong/1-8/1968(H3N2) | 3 (PA) | 2181 | CY034001 |
| A/Hong Kong/1-9/1968(H3N2) | 3 (PA) | 2191 | CY033054 |
| A/Hong Kong/1-11/1968(H3N2) | 3 (PA) | 2191 | CY034009 |
| A/Hong Kong/1-12/1968(H3N2) | 3 (PA) | 2182 | CY033070 |
| A/Hong Kong/1-1-MA-12/1968(H3N2) | 3 (PA) | 2182 | CY033510 |
| A/Hong Kong/1-1-MA-12A/1968(H3N2) | 3 (PA) | 2166 | CY034947 |
| A/Hong Kong/1-1-MA-12B/1968(H3N2) | 3 (PA) | 2181 | CY034955 |
| A/Hong Kong/1-1-MA-12C/1968(H3N2) | 3 (PA) | 2166 | CY034963 |
| A/Hong Kong/1-1-MA-12D/1968(H3N2) | 3 (PA) | 2170 | CY033993 |
| A/Hong Kong/1-1-MA-12E/1968(H3N2) | 3 (PA) | 2170 | CY034971 |
| A/Hong Kong/1-1-MA-20/1968(H3N2) | 3 (PA) | 2177 | CY044274 |
| A/Hong Kong/1-1-MA-20A/1968(H3N2) | 3 (PA) | 2170 | CY032982 |
| A/Hong Kong/1-1-MA-20B/1968(H3N2) | 3 (PA) | 2181 | CY032990 |
| A/Hong Kong/1-1-MA-20C/1968(H3N2) | 3 (PA) | 2182 | CY045721 |
| A/Hong Kong/1-1-MA-20D/1968(H3N2) | 3 (PA) | 2178 | CY034979 |
| A/Hong Kong/1-1-MA-20E/1968(H3N2) | 3 (PA) | 2189 | CY032998 |
| A/Hong Kong/1-1-MA21-1/1968(H3N2) | 3 (PA) | 2191 | CY033078 |
| A/Hong Kong/1-1-MA21-2/1968(H3N2) | 3 (PA) | 2189 | CY033086 |
| A/Hong Kong/1-1-MA21-3/1968(H3N2) | 3 (PA) | 2191 | CY033094 |
| A/Hong Kong/1-2-MA21-1/1968(H3N2) | 3 (PA) | 2190 | CY033102 |
| A/Hong Kong/1-2-MA21-2/1968(H3N2) | 3 (PA) | 2191 | CY033110 |
| A/Hong Kong/1-2-MA21-3/1968(H3N2) | 3 (PA) | 2209 | HM641162 |
| A/Hong Kong/1-4-MA21-1/1968(H3N2) | 3 (PA) | 2209 | HM641161 |
| A/Hong Kong/1-4-MA21-2/1968(H3N2) | 3 (PA) | 2176 | CY033118 |
| A/Hong Kong/1-4-MA21-3/1968(H3N2) | 3 (PA) | 2191 | CY033126 |
| A/Hong Kong/1-5-MA21-1/1968(H3N2) | 3 (PA) | 2209 | HM641160 |
| A/Hong Kong/1-5-MA21-2/1968(H3N2) | 3 (PA) | 2191 | CY033134 |
| A/Hong Kong/1-5-MA21-3/1968(H3N2) | 3 (PA) | 2166 | CY044282 |
| A/Hong Kong/1-6-MA21-1/1968(H3N2) | 3 (PA) | 2169 | CY044290 |
| A/Hong Kong/1-6-MA21-2/1968(H3N2) | 3 (PA) | 2189 | CY034017 |
| A/Hong Kong/1-6-MA21-3/1968(H3N2) | 3 (PA) | 2166 | CY045737 |
| A/Hong Kong/1-8-MA21-1/1968(H3N2) | 3 (PA) | 2190 | CY034025 |
| A/Hong Kong/1-8-MA21-2/1968(H3N2) | 3 (PA) | 2186 | CY033142 |
| A/Hong Kong/1-8-MA21-3/1968(H3N2) | 3 (PA) | 2183 | CY034033 |
| A/Hong Kong/1-9-MA21-1/1968(H3N2) | 3 (PA) | 2191 | CY033518 |
| A/Hong Kong/1-9-MA21-2/1968(H3N2) | 3 (PA) | 2191 | CY033526 |
| A/Hong Kong/1-9-MA21-3/1968(H3N2) | 3 (PA) | 2192 | CY033534 |
| A/Hong Kong/1-11-MA21-1/1968(H3N2) | 3 (PA) | 2190 | CY033542 |
| A/Hong Kong/1-11-MA21-2/1968(H3N2) | 3 (PA) | 2190 | CY033550 |
| A/Hong Kong/1-11-MA21-3/1968(H3N2) | 3 (PA) | 2189 | CY033558 |
| A/Hong Kong/1-12-MA21-1/1968(H3N2) | 3 (PA) | 2192 | CY033566 |
| A/Hong Kong/1-12-MA21-2/1968(H3N2) | 3 (PA) | 2192 | CY033574 |
| A/Hong Kong/1-12-MA21-3/1968(H3N2) | 3 (PA) | 2158 | CY034041 |
|  |  |  |  |
| Virus clone name | segment (protein) | nucleotides | Accession No. |
| A/Hong Kong/1/1968(H3N2) | 5 (NP) | 1506 | CY044264 |
| A/Hong Kong/1-1/1968(H3N2) | 5 (NP) | 1519 | CY033004 |
| A/Hong Kong/1-2/1968(H3N2) | 5 (NP) | 1519 | CY033012 |
| A/Hong Kong/1-4/1968(H3N2) | 5 (NP) | 1518 | CY033020 |
| A/Hong Kong/1-5/1968(H3N2) | 5 (NP) | 1522 | CY033028 |
| A/Hong Kong/1-6/1968(H3N2) | 5 (NP) | 1522 | CY033036 |
| A/Hong Kong/1-8/1968(H3N2) | 5 (NP) | 1522 | CY033999 |
| A/Hong Kong/1-9/1968(H3N2) | 5 (NP) | 1522 | CY033052 |
| A/Hong Kong/1-11/1968(H3N2) | 5 (NP) | 1516 | CY034007 |
| A/Hong Kong/1-12/1968(H3N2) | 5 (NP) | 1518 | CY033068 |
| A/Hong Kong/1-1-MA-12/1968(H3N2) | 5 (NP) | 1512 | CY033508 |
| A/Hong Kong/1-1-MA-12A/1968(H3N2) | 5 (NP) | 1523 | CY034945 |
| A/Hong Kong/1-1-MA-12B/1968(H3N2) | 5 (NP) | 1512 | CY034953 |
| A/Hong Kong/1-1-MA-12C/1968(H3N2) | 5 (NP) | 1510 | CY034961 |
| A/Hong Kong/1-1-MA-12D/1968(H3N2) | 5 (NP) | 1503 | CY033991 |
| A/Hong Kong/1-1-MA-12E/1968(H3N2) | 5 (NP) | 1514 | CY034969 |
| A/Hong Kong/1-1-MA-20/1968(H3N2) | 5 (NP) | 1498 | CY044272 |
| A/Hong Kong/1-1-MA-20A/1968(H3N2) | 5 (NP) | 1518 | CY032980 |
| A/Hong Kong/1-1-MA-20B/1968(H3N2) | 5 (NP) | 1522 | CY032988 |
| A/Hong Kong/1-1-MA-20C/1968(H3N2) | 5 (NP) | 1520 | CY045719 |
| A/Hong Kong/1-1-MA-20D/1968(H3N2) | 5 (NP) | 1516 | CY034977 |
| A/Hong Kong/1-1-MA-20E/1968(H3N2) | 5 (NP) | 1518 | CY032996 |
| A/Hong Kong/1-1-MA21-1/1968(H3N2) | 5 (NP) | 1519 | CY033076 |
| A/Hong Kong/1-1-MA21-2/1968(H3N2) | 5 (NP) | 1518 | CY033084 |
| A/Hong Kong/1-1-MA21-3/1968(H3N2) | 5 (NP) | 1523 | CY033092 |
| A/Hong Kong/1-2-MA21-1/1968(H3N2) | 5 (NP) | 1518 | CY033100 |
| A/Hong Kong/1-2-MA21-2/1968(H3N2) | 5 (NP) | 1523 | CY033108 |
| A/Hong Kong/1-2-MA21-3/1968(H3N2) | 5 (NP) | 1520 | HM641184 |
| A/Hong Kong/1-4-MA21-1/1968(H3N2) | 5 (NP) | 1520 | HM641183 |
| A/Hong Kong/1-4-MA21-2/1968(H3N2) | 5 (NP) | 1517 | CY033116 |
| A/Hong Kong/1-4-MA21-3/1968(H3N2) | 5 (NP) | 1522 | CY033124 |
| A/Hong Kong/1-5-MA21-1/1968(H3N2) | 5 (NP) | 1520 | HM641182 |
| A/Hong Kong/1-5-MA21-2/1968(H3N2) | 5 (NP) | 1522 | CY033132 |
| A/Hong Kong/1-5-MA21-3/1968(H3N2) | 5 (NP) | 1497 | CY044280 |
| A/Hong Kong/1-6-MA21-1/1968(H3N2) | 5 (NP) | 1503 | CY044288 |
| A/Hong Kong/1-6-MA21-2/1968(H3N2) | 5 (NP) | 1519 | CY034015 |
| A/Hong Kong/1-6-MA21-3/1968(H3N2) | 5 (NP) | 1503 | CY045735 |
| A/Hong Kong/1-8-MA21-1/1968(H3N2) | 5 (NP) | 1504 | CY034023 |
| A/Hong Kong/1-8-MA21-2/1968(H3N2) | 5 (NP) | 1520 | CY033140 |
| A/Hong Kong/1-8-MA21-3/1968(H3N2) | 5 (NP) | 1518 | CY034031 |
| A/Hong Kong/1-9-MA21-1/1968(H3N2) | 5 (NP) | 1520 | CY033516 |
| A/Hong Kong/1-9-MA21-2/1968(H3N2) | 5 (NP) | 1525 | CY033524 |
| A/Hong Kong/1-9-MA21-3/1968(H3N2) | 5 (NP) | 1519 | CY033532 |
| A/Hong Kong/1-11-MA21-1/1968(H3N2) | 5 (NP) | 1520 | CY033540 |
| A/Hong Kong/1-11-MA21-2/1968(H3N2) | 5 (NP) | 1519 | CY033548 |
| A/Hong Kong/1-11-MA21-3/1968(H3N2) | 5 (NP) | 1519 | CY033556 |
| A/Hong Kong/1-12-MA21-1/1968(H3N2) | 5 (NP) | 1522 | CY033564 |
| A/Hong Kong/1-12-MA21-2/1968(H3N2) | 5 (NP) | 1520 | CY033572 |
| A/Hong Kong/1-12-MA21-3/1968(H3N2) | 5 (NP) | 1504 | CY034039 |
|  |  |  |  |
| Virus clone name | segment (protein) | nucleotides | Accession No. |
| A/Hong Kong/1/1968(H3N2) | 4 (HA) | 1717 | CY044261 |
| A/Hong Kong/1-1/1968(H3N2) | 4 (HA) | 1719 | CY033001 |
| A/Hong Kong/1-2/1968(H3N2) | 4 (HA) | 1719 | CY033009 |
| A/Hong Kong/1-4/1968(H3N2) | 4 (HA) | 1720 | CY033017 |
| A/Hong Kong/1-5/1968(H3N2) | 4 (HA) | 1720 | CY033025 |
| A/Hong Kong/1-6/1968(H3N2) | 4 (HA) | 1719 | CY033033 |
| A/Hong Kong/1-8/1968(H3N2) | 4 (HA) | 1719 | CY033996 |
| A/Hong Kong/1-9/1968(H3N2) | 4 (HA) | 1719 | CY033049 |
| A/Hong Kong/1-11/1968(H3N2) | 4 (HA) | 1712 | CY034004 |
| A/Hong Kong/1-12/1968(H3N2) | 4 (HA) | 1714 | CY033065 |
| A/Hong Kong/1-1-MA-12/1968(H3N2) | 4 (HA) | 1714 | CY033505 |
| A/Hong Kong/1-1-MA-12A/1968(H3N2) | 4 (HA) | 1716 | CY034942 |
| A/Hong Kong/1-1-MA-12B/1968(H3N2) | 4 (HA) | 1714 | CY034950 |
| A/Hong Kong/1-1-MA-12C/1968(H3N2) | 4 (HA) | 1719 | CY034958 |
| A/Hong Kong/1-1-MA-12D/1968(H3N2) | 4 (HA) | 1719 | CY033988 |
| A/Hong Kong/1-1-MA-12E/1968(H3N2)) | 4 (HA) | 1721 | CY034966 |
| A/Hong Kong/1-1-MA-20/1968(H3N2) | 4 (HA) | 1716 | CY044269 |
| A/Hong Kong/1-1-MA-20A/1968(H3N2) | 4 (HA) | 1722 | CY032977 |
| A/Hong Kong/1-1-MA-20B/1968(H3N2) | 4 (HA) | 1725 | CY032985 |
| A/Hong Kong/1-1-MA-20C/1968(H3N2) | 4 (HA) | 1721 | CY045716 |
| A/Hong Kong/1-1-MA-20D/1968(H3N2) | 4 (HA) | 1713 | CY034974 |
| A/Hong Kong/1-1-MA-20E/1968(H3N2) | 4 (HA) | 1716 | CY032993 |
| A/Hong Kong/1-1-MA21-1/1968(H3N2) | 4 (HA) | 1714 | CY033073 |
| A/Hong Kong/1-1-MA21-2/1968(H3N2) | 4 (HA) | 1717 | CY033081 |
| A/Hong Kong/1-1-MA21-3/1968(H3N2) | 4 (HA) | 1722 | CY033089 |
| A/Hong Kong/1-2-MA21-1/1968(H3N2) | 4 (HA) | 1722 | CY033097 |
| A/Hong Kong/1-2-MA21-2/1968(H3N2) | 4 (HA) | 1716 | CY033105 |
| A/Hong Kong/1-2-MA21-3/1968(H3N2) | 4 (HA) | 1736 | HM641173 |
| A/Hong Kong/1-4-MA21-1/1968(H3N2) | 4 (HA) | 1736 | HM641172 |
| A/Hong Kong/1-4-MA21-2/1968(H3N2) | 4 (HA) | 1713 | CY033113 |
| A/Hong Kong/1-4-MA21-3/1968(H3N2) | 4 (HA) | 1713 | CY033121 |
| A/Hong Kong/1-5-MA21-1/1968(H3N2) | 4 (HA) | 1736 | HM641171 |
| A/Hong Kong/1-5-MA21-2/1968(H3N2) | 4 (HA) | 1707 | CY033129 |
| A/Hong Kong/1-5-MA21-3/1968(H3N2) | 4 (HA) | 1705 | CY044277 |
| A/Hong Kong/1-6-MA21-1/1968(H3N2) | 4 (HA) | 1707 | CY044285 |
| A/Hong Kong/1-6-MA21-2/1968(H3N2) | 4 (HA) | 1715 | CY034012 |
| A/Hong Kong/1-6-MA21-3/1968(H3N2) | 4 (HA) | 1710 | CY045732 |
| A/Hong Kong/1-8-MA21-1/1968(H3N2) | 4 (HA) | 1724 | CY034020 |
| A/Hong Kong/1-8-MA21-2/1968(H3N2) | 4 (HA) | 1719 | CY033137 |
| A/Hong Kong/1-8-MA21-3/1968(H3N2) | 4 (HA) | 1717 | CY034028 |
| A/Hong Kong/1-9-MA21-1/1968(H3N2) | 4 (HA) | 1719 | CY033513 |
| A/Hong Kong/1-9-MA21-2/1968(H3N2) | 4 (HA) | 1719 | CY033521 |
| A/Hong Kong/1-9-MA21-3/1968(H3N2) | 4 (HA) | 1726 | CY033529 |
| A/Hong Kong/1-11-MA21-1/1968(H3N2) | 4 (HA) | 1715 | CY033537 |
| A/Hong Kong/1-11-MA21-2/1968(H3N2) | 4 (HA) | 1719 | CY033545 |
| A/Hong Kong/1-11-MA21-3/1968(H3N2) | 4 (HA) | 1719 | CY033553 |
| A/Hong Kong/1-12-MA21-1/1968(H3N2) | 4 (HA) | 1719 | CY033561 |
| A/Hong Kong/1-12-MA21-2/1968(H3N2) | 4 (HA) | 1719 | CY033569 |
| A/Hong Kong/1-12-MA21-3/1968(H3N2) | 4 (HA) | 1716 | CY034036 |
|  |  |  |  |
| Virus clone name | segment (protein) | nucleotides | Accession No. |
| A/Hong Kong/1/1968(H3N2) | 6 (NA) | 1420 | CY044263 |
| A/Hong Kong/1-1/1968(H3N2) | 6 (NA) | 1417 | CY033003 |
| A/Hong Kong/1-2/1968(H3N2) | 6 (NA) | 1416 | CY033011 |
| A/Hong Kong/1-4/1968(H3N2) | 6 (NA) | 1419 | CY033019 |
| A/Hong Kong/1-5/1968(H3N2) | 6 (NA) | 1430 | CY033027 |
| A/Hong Kong/1-6/1968(H3N2) | 6 (NA) | 1431 | CY033035 |
| A/Hong Kong/1-8/1968(H3N2) | 6 (NA) | 1430 | CY033998 |
| A/Hong Kong/1-9/1968(H3N2) | 6 (NA) | 1431 | CY033051 |
| A/Hong Kong/1-11/1968(H3N2) | 6 (NA) | 1415 | CY034006 |
| A/Hong Kong/1-12/1968(H3N2) | 6 (NA) | 1415 | CY033067 |
| A/Hong Kong/1-1-MA-12/1968(H3N2) | 6 (NA) | 1411 | CY033507 |
| A/Hong Kong/1-1-MA-12A/1968(H3N2) | 6 (NA) | 1414 | CY034944 |
| A/Hong Kong/1-1-MA-12B/1968(H3N2) | 6 (NA) | 1426 | CY034952 |
| A/Hong Kong/1-1-MA-12C/1968(H3N2) | 6 (NA) | 1420 | CY034960 |
| A/Hong Kong/1-1-MA-12D/1968(H3N2) | 6 (NA) | 1426 | CY033990 |
| A/Hong Kong/1-1-MA-12E/1968(H3N2) | 6 (NA) | 1425 | CY034968 |
| A/Hong Kong/1-1-MA-20/1968(H3N2) | 6 (NA) | 1425 | CY044271 |
| A/Hong Kong/1-1-MA-20A/1968(H3N2) | 6 (NA) | 1420 | CY032979 |
| A/Hong Kong/1-1-MA-20B/1968(H3N2) | 6 (NA) | 1414 | CY032987 |
| A/Hong Kong/1-1-MA-20C/1968(H3N2) | 6 (NA) | 1426 | CY045718 |
| A/Hong Kong/1-1-MA-20D/1968(H3N2) | 6 (NA) | 1426 | CY034976 |
| A/Hong Kong/1-1-MA-20E/1968(H3N2) | 6 (NA) | 1421 | CY032995 |
| A/Hong Kong/1-1-MA21-1/1968(H3N2) | 6 (NA) | 1412 | CY033075 |
| A/Hong Kong/1-1-MA21-2/1968(H3N2) | 6 (NA) | 1430 | CY033083 |
| A/Hong Kong/1-1-MA21-3/1968(H3N2) | 6 (NA) | 1429 | CY033091 |
| A/Hong Kong/1-2-MA21-1/1968(H3N2) | 6 (NA) | 1420 | CY033099 |
| A/Hong Kong/1-2-MA21-2/1968(H3N2) | 6 (NA) | 1420 | CY033107 |
| A/Hong Kong/1-2-MA21-3/1968(H3N2) | 6 (NA) | 1467 | HM641195 |
| A/Hong Kong/1-4-MA21-1/1968(H3N2) | 6 (NA) | 1467 | HM641194 |
| A/Hong Kong/1-4-MA21-2/1968(H3N2) | 6 (NA) | 1432 | CY033115 |
| A/Hong Kong/1-4-MA21-3/1968(H3N2) | 6 (NA) | 1422 | CY033123 |
| A/Hong Kong/1-5-MA21-1/1968(H3N2) | 6 (NA) | 1467 | HM641193 |
| A/Hong Kong/1-5-MA21-2/1968(H3N2) | 6 (NA) | 1431 | CY033131 |
| A/Hong Kong/1-5-MA21-3/1968(H3N2) | 6 (NA) | 1432 | CY044279 |
| A/Hong Kong/1-6-MA21-1/1968(H3N2) | 6 (NA) | 1416 | CY044287 |
| A/Hong Kong/1-6-MA21-2/1968(H3N2) | 6 (NA) | 1420 | CY034014 |
| A/Hong Kong/1-6-MA21-3/1968(H3N2) | 6 (NA) | 1416 | CY045734 |
| A/Hong Kong/1-8-MA21-1/1968(H3N2) | 6 (NA) | 1432 | CY034022 |
| A/Hong Kong/1-8-MA21-2/1968(H3N2) | 6 (NA) | 1420 | CY033139 |
| A/Hong Kong/1-8-MA21-3/1968(H3N2) | 6 (NA) | 1410 | CY034030 |
| A/Hong Kong/1-9-MA21-1/1968(H3N2) | 6 (NA) | 1416 | CY033515 |
| A/Hong Kong/1-9-MA21-2/1968(H3N2) | 6 (NA) | 1423 | CY033523 |
| A/Hong Kong/1-9-MA21-3/1968(H3N2) | 6 (NA) | 1429 | CY033531 |
| A/Hong Kong/1-11-MA21-1/1968(H3N2) | 6 (NA) | 1430 | CY033539 |
| A/Hong Kong/1-11-MA21-2/1968(H3N2) | 6 (NA) | 1430 | CY033547 |
| A/Hong Kong/1-11-MA21-3/1968(H3N2) | 6 (NA) | 1430 | CY033555 |
| A/Hong Kong/1-12-MA21-1/1968(H3N2) | 6 (NA) | 1431 | CY033563 |
| A/Hong Kong/1-12-MA21-2/1968(H3N2) | 6 (NA) | 1430 | CY033571 |
| A/Hong Kong/1-12-MA21-3/1968(H3N2) | 6 (NA) | 1414 | CY034038 |
|  |  |  |  |
| Virus clone name | segment (protein) | nucleotides | Accession No. |
| A/Hong Kong/1/1968(H3N2) | 7 (M1/M2) | 985 | CY044262 |
| A/Hong Kong/1-1/1968(H3N2) | 7(M1/M2) | 983 | CY033002 |
| A/Hong Kong/1-2/1968(H3N2) | 7 (M1/M2) | 983 | CY033010 |
| A/Hong Kong/1-4/1968(H3N2) | 7 (M1/M2) | 982 | CY033018 |
| A/Hong Kong/1-5/1968(H3N2) | 7 (M1/M2) | 982 | CY033026 |
| A/Hong Kong/1-6/1968(H3N2) | 7 (M1/M2) | 983 | CY033034 |
| A/Hong Kong/1-8/1968(H3N2) | 7 (M1/M2) | 984 | CY033997 |
| A/Hong Kong/1-9/1968(H3N2) | 7 (M1/M2) | 985 | CY033050 |
| A/Hong Kong/1-11/1968(H3N2) | 7 (M1/M2) | 985 | CY034005 |
| A/Hong Kong/1-12/1968(H3N2) | 7 (M1/M2) | 985 | CY033066 |
| A/Hong Kong/1-1-MA-12/1968(H3N2) | 7 (M1/M2) | 982 | CY033506 |
| A/Hong Kong/1-1-MA-12A/1968(H3N2) | 7 (M1/M2) | 983 | CY034943 |
| A/Hong Kong/1-1-MA-12B/1968(H3N2) | 7 (M1/M2) | 983 | CY034951 |
| A/Hong Kong/1-1-MA-12C/1968(H3N2) | 7 (M1/M2) | 985 | CY034959 |
| A/Hong Kong/1-1-MA-12D/1968(H3N2) | 7 (M1/M2) | 983 | CY033989 |
| A/Hong Kong/1-1-MA-12E/1968(H3N2) | 7 (M1/M2) | 982 | CY034967 |
| A/Hong Kong/1-1-MA-20/1968(H3N2) | 7 (M1/M2) | 985 | CY044270 |
| A/Hong Kong/1-1-MA-20A/1968(H3N2) | 7 (M1/M2) | 984 | CY032978 |
| A/Hong Kong/1-1-MA-20B/1968(H3N2) | 7 (M1/M2) | 985 | CY032986 |
| A/Hong Kong/1-1-MA-20C/1968(H3N2) | 7 (M1/M2) | 986 | CY045717 |
| A/Hong Kong/1-1-MA-20D/1968(H3N2) | 7 (M1/M2) | 985 | CY034975 |
| A/Hong Kong/1-1-MA-20E/1968(H3N2) | 7 (M1/M2) | 983 | CY032994 |
| A/Hong Kong/1-1-MA21-1/1968(H3N2) | 7 (M1/M2) | 984 | CY033074 |
| A/Hong Kong/1-1-MA21-2/1968(H3N2) | 7 (M1/M2) | 983 | CY033082 |
| A/Hong Kong/1-1-MA21-3/1968(H3N2) | 7 (M1/M2) | 984 | CY033090 |
| A/Hong Kong/1-2-MA21-1/1968(H3N2) | 7 (M1/M2) | 984 | CY033098 |
| A/Hong Kong/1-2-MA21-2/1968(H3N2) | 7 (M1/M2) | 985 | CY033106 |
| A/Hong Kong/1-2-MA21-3/1968(H3N2) | 7 (M1/M2) | 1002 | HM641206 |
| A/Hong Kong/1-4-MA21-1/1968(H3N2) | 7 (M1/M2) | 1002 | HM641205 |
| A/Hong Kong/1-4-MA21-2/1968(H3N2) | 7 (M1/M2) | 985 | CY033114 |
| A/Hong Kong/1-4-MA21-3/1968(H3N2) | 7 (M1/M2) | 985 | CY033122 |
| A/Hong Kong/1-5-MA21-1/1968(H3N2) | 7 (M1/M2) | 1002 | HM641204 |
| A/Hong Kong/1-5-MA21-2/1968(H3N2) | 7 (M1/M2) | 984 | CY033130 |
| A/Hong Kong/1-5-MA21-3/1968(H3N2) | 7 (M1/M2) | 985 | CY044278 |
| A/Hong Kong/1-6-MA21-1/1968(H3N2) | 7 (M1/M2) | 985 | CY044286 |
| A/Hong Kong/1-6-MA21-2/1968(H3N2) | 7 (M1/M2) | 985 | CY034013 |
| A/Hong Kong/1-6-MA21-3/1968(H3N2) | 7 (M1/M2) | 985 | CY045733 |
| A/Hong Kong/1-8-MA21-1/1968(H3N2) | 7 (M1/M2) | 984 | CY034021 |
| A/Hong Kong/1-8-MA21-2/1968(H3N2) | 7 (M1/M2) | 985 | CY033138 |
| A/Hong Kong/1-8-MA21-3/1968(H3N2) | 7 (M1/M2) | 984 | CY034029 |
| A/Hong Kong/1-9-MA21-1/1968(H3N2) | 7 (M1/M2) | 985 | CY033514 |
| A/Hong Kong/1-9-MA21-2/1968(H3N2) | 7 (M1/M2) | 983 | CY033522 |
| A/Hong Kong/1-9-MA21-3/1968(H3N2) | 7 (M1/M2) | 986 | CY033530 |
| A/Hong Kong/1-11-MA21-1/1968(H3N2) | 7 (M1/M2) | 985 | CY033538 |
| A/Hong Kong/1-11-MA21-2/1968(H3N2) | 7 (M1/M2) | 984 | CY033546 |
| A/Hong Kong/1-11-MA21-3/1968(H3N2) | 7 (M1/M2) | 985 | CY033554 |
| A/Hong Kong/1-12-MA21-1/1968(H3N2) | 7 (M1/M2) | 985 | CY033562 |
| A/Hong Kong/1-12-MA21-2/1968(H3N2) | 7 (M1/M2) | 985 | CY033570 |
| A/Hong Kong/1-12-MA21-3/1968(H3N2) | 7 (M1/M2) | 983 | CY034037 |
|  |  |  |  |
| Virus clone name | segment (protein) | nucleotides | Accession No. |
| A/Hong Kong/1/1968(H3N2) | 8 (NS1/NEP) | 847 | CY044265 |
| A/Hong Kong/1-1/1968(H3N2) | 8 (NS1/NEP) | 845 | CY033005 |
| A/Hong Kong/1-2/1968(H3N2) | 8 (NS1/NEP) | 845 | CY033013 |
| A/Hong Kong/1-4/1968(H3N2) | 8 (NS1/NEP) | 845 | CY033021 |
| A/Hong Kong/1-5/1968(H3N2) | 8 (NS1/NEP) | 845 | CY033029 |
| A/Hong Kong/1-6/1968(H3N2) | 8 (NS1/NEP) | 844 | CY033037 |
| A/Hong Kong/1-8/1968(H3N2) | 8 (NS1/NEP) | 847 | CY034000 |
| A/Hong Kong/1-9/1968(H3N2) | 8 (NS1/NEP) | 845 | CY033053 |
| A/Hong Kong/1-11/1968(H3N2) | 8 (NS1/NEP) | 844 | CY034008 |
| A/Hong Kong/1-12/1968(H3N2) | 8 (NS1/NEP) | 842 | CY033069 |
| A/Hong Kong/1-1-MA-12/1968(H3N2) | 8 (NS1/NEP) | 846 | CY033509 |
| A/Hong Kong/1-1-MA-12A/1968(H3N2) | 8 (NS1/NEP) | 845 | CY034946 |
| A/Hong Kong/1-1-MA-12B/1968(H3N2) | 8 (NS1/NEP) | 850 | CY034954 |
| A/Hong Kong/1-1-MA-12C/1968(H3N2) | 8 (NS1/NEP) | 845 | CY034962 |
| A/Hong Kong/1-1-MA-12D/1968(H3N2) | 8 (NS1/NEP) | 845 | CY033992 |
| A/Hong Kong/1-1-MA-12E/1968(H3N2) | 8 (NS1/NEP) | 847 | CY034970 |
| A/Hong Kong/1-1-MA-20/1968(H3N2) | 8 (NS1/NEP) | 848 | CY044273 |
| A/Hong Kong/1-1-MA-20A/1968(H3N2) | 8 (NS1/NEP) | 847 | CY032981 |
| A/Hong Kong/1-1-MA-20B/1968(H3N2) | 8 (NS1/NEP) | 846 | CY032989 |
| A/Hong Kong/1-1-MA-20C/1968(H3N2) | 8 (NS1/NEP) | 848 | CY045720 |
| A/Hong Kong/1-1-MA-20D/1968(H3N2) | 8 (NS1/NEP) | 847 | CY034978 |
| A/Hong Kong/1-1-MA-20E/1968(H3N2) | 8 (NS1/NEP) | 847 | CY032997 |
| A/Hong Kong/1-1-MA21-1/1968(H3N2) | 8 (NS1/NEP) | 846 | CY033077 |
| A/Hong Kong/1-1-MA21-2/1968(H3N2) | 8 (NS1/NEP) | 846 | CY033085 |
| A/Hong Kong/1-1-MA21-3/1968(H3N2) | 8 (NS1/NEP) | 845 | CY033093 |
| A/Hong Kong/1-2-MA21-1/1968(H3N2) | 8 (NS1/NEP) | 847 | CY033101 |
| A/Hong Kong/1-2-MA21-2/1968(H3N2) | 8 (NS1/NEP) | 847 | CY033109 |
| A/Hong Kong/1-2-MA21-3/1968(H3N2) | 8 (NS1/NEP) | 890 | HM641217 |
| A/Hong Kong/1-4-MA21-1/1968(H3N2) | 8 (NS1/NEP) | 890 | HM641216 |
| A/Hong Kong/1-4-MA21-2/1968(H3N2) | 8 (NS1/NEP) | 845 | CY033117 |
| A/Hong Kong/1-4-MA21-3/1968(H3N2) | 8 (NS1/NEP) | 844 | CY033125 |
| A/Hong Kong/1-5-MA21-1/1968(H3N2) | 8 (NS1/NEP) | 890 | HM641215 |
| A/Hong Kong/1-5-MA21-2/1968(H3N2) | 8 (NS1/NEP) | 845 | CY033133 |
| A/Hong Kong/1-5-MA21-3/1968(H3N2) | 8 (NS1/NEP) | 847 | CY044281 |
| A/Hong Kong/1-6-MA21-1/1968(H3N2) | 8 (NS1/NEP) | 838 | CY044289 |
| A/Hong Kong/1-6-MA21-2/1968(H3N2) | 8 (NS1/NEP) | 846 | CY034016 |
| A/Hong Kong/1-6-MA21-3/1968(H3N2) | 8 (NS1/NEP) | 847 | CY045736 |
| A/Hong Kong/1-8-MA21-1/1968(H3N2) | 8 (NS1/NEP) | 847 | CY034024 |
| A/Hong Kong/1-8-MA21-2/1968(H3N2) | 8 (NS1/NEP) | 848 | CY033141 |
| A/Hong Kong/1-8-MA21-3/1968(H3N2) | 8 (NS1/NEP) | 847 | CY034032 |
| A/Hong Kong/1-9-MA21-1/1968(H3N2) | 8 (NS1/NEP) | 845 | CY033517 |
| A/Hong Kong/1-9-MA21-2/1968(H3N2) | 8 (NS1/NEP) | 846 | CY033525 |
| A/Hong Kong/1-9-MA21-3/1968(H3N2) | 8 (NS1/NEP) | 848 | CY033533 |
| A/Hong Kong/1-11-MA21-1/1968(H3N2) | 8 (NS1/NEP) | 847 | CY033541 |
| A/Hong Kong/1-11-MA21-2/1968(H3N2) | 8 (NS1/NEP) | 847 | CY033549 |
| A/Hong Kong/1-11-MA21-3/1968(H3N2) | 8 (NS1/NEP) | 847 | CY033557 |
| A/Hong Kong/1-12-MA21-1/1968(H3N2) | 8 (NS1/NEP) | 847 | CY033565 |
| A/Hong Kong/1-12-MA21-2/1968(H3N2) | 8 (NS1/NEP) | 848 | CY033573 |
| A/Hong Kong/1-12-MA21-3/1968(H3N2) | 8 (NS1/NEP) | 845 | CY034040 |
