# Supplementary material for: Genomic and Protein Structural Maps of Adaptive Evolution of Human Influenza A Virus to Increased Virulence in the Mouse
Source: PLoS One. 2011 Jun 30;6(6):e21740. doi: 10.1371/journal.pone.0021740 (PMC3128085; doi:10.1371/journal.pone.0021740)
Supplement: Table S10 — List of PB2 gene Genbank accession numbers for human H5N1 and canine H3N8 isolates that possess PB2 D701N and/or PB2 D740N mutations. (DOC) [file pone.0021740.s010.doc]

**Table S10. List of PB2 gene Genbank accession numbers for human H5N1 and canine H3N8 isolates that possess PB2 D701N and/or PB2 D740N mutations.**

AAK49380 **PB2 D701N** A/HongKong/97/98(H5N1)

ADF83735 **PB2 D701N** A/Vietnam/UT3030/2003(H5N1)

AAV35117 **PB2 D701N** A/Thailand/1(KAN-1)/2004(H5N1)

ADF83731 **PB2 D701N** A/Vietnam/UT3047III/2004(H5N1)

ADF83728 **PB2 D701N** A/Vietnam/HN30262IIIM3/2004(H5N1)

ABF01749 **PB2 D701N** A/Vietnam/CL115/2005(H5N1)

ABW06307 **PB2 D740N** A/Indonesia/245H/2005(H5N1)

ABI36227^a^ **PB2 D740N** A/Indonesia/CDC292N/2005(H5N1)

ABI36228^a^ **PB2 D740N** A/Indonesia/CDC292T/2005(H5N1)

ABI36432 **PB2 D740N** A/Indonesia/CDC669/2006(H5N1)

ADF83720 **PB2 D740N** A/Vietnam/HN31388M1/2007(H5N1)

[ABY81502](http://www.ncbi.nlm.nih.gov/entrez/viewer.fcgi?val=ABY81502) **PB2 D701N** A/equine/Miami/1/1963(H3N8) (prototype)

[ADM29316](http://www.ncbi.nlm.nih.gov/entrez/viewer.fcgi?val=ADM29316) **PB2 D701N+D740N** A/canine/New York/5183-6/2006(H3N8)

[ADM29382](http://www.ncbi.nlm.nih.gov/entrez/viewer.fcgi?val=ADM29382) **PB2 D701N+D740N** (A/canine/Colorado/30604/2006(H3N8)

[ADM29679](http://www.ncbi.nlm.nih.gov/entrez/viewer.fcgi?val=ADM29679) **PB2 D701N+D740N** (A/canine/New York/145353/2008(H3N8)

a, samples from the trachea and nasal tract of the same patient

accession numbers obtained from alignments of 154 human H5N1 and 37 canine H3N8 PB2 genes at the National Center for Biotechnology Information, http://www.ncbi.nlm.nih.gov/genomes/FLU/on March 30, 2011.
