# Supplementary material for: Genomic and Protein Structural Maps of Adaptive Evolution of Human Influenza A Virus to Increased Virulence in the Mouse
Source: PLoS One. 2011 Jun 30;6(6):e21740. doi: 10.1371/journal.pone.0021740 (PMC3128085; doi:10.1371/journal.pone.0021740)
Supplement: Figure S2 — Blast alignment of mouse CPSF30 with human CPSF30. Amino acid sequence of mouse CPSF30 (query) is aligned above human CPSF30 sequence (Sbjct) with the consensus sequence indicated between each sequence. The F2F3 binding fragment is indicated in yellow mask showing identical amino acid sequence between human and mouse. (PDF) [file pone.0021740.s012.pdf]

Blast Description

gi|183229548|gb|ACC60272.1| cleavage and polyadenylation specific factor 4 isoform 2 [Mus musculus]Query Length 244
>CPSF-F2F3 fragment is masked in yellow

>ref|NP_001075028.1| cleavage and polyadenylation specificity factor subunit 4 isoform 2 [Homo sapiens]

GENE ID: 10898 CPSF4 | cleavage and polyadenylation specific factor 4, 30kDa
[Homo sapiens] (Over 10 PubMed links)

Score = 503 bits (1296), Expect = 5e-141, Method: Compositional matrix adjust.
Identities = 242/244 (99%), Positives = 242/244 (99%), Gaps = 0/244 (0%)

```
Query  1    MQEIIASVDHIKFDLEIAVEQQLGAQPLPFPGMDKSGAAVCEFFLKAACGKGGMCPFRHI  60
            MQEIIASVDHIKFDLEIAVEQQLGAQPLPFPGMDKSGAAVCEFFLKAACGKGGMCPFRHI
Sbjct  1    MQEIIASVDHIKFDLEIAVEQQLGAQPLPFPGMDKSGAAVCEFFLKAACGKGGMCPFRHI  60

Query  61   SGEKTVVCKHWLRGLCKKGDQCEFLHEYDMTKMPECYFYSKFGECSNKECPFLHIDPESK  120
            SGEKTVVCKHWLRGLCKKGDQCEFLHEYDMTKMPECYFYSKFGECSNKECPFLHIDPESK
Sbjct  61   SGEKTVVCKHWLRGLCKKGDQCEFLHEYDMTKMPECYFYSKFGECSNKECPFLHIDPESK  120

Query  121  IKDCPWYDRGFCKHGPLCRHRHTRRVICVNYLVGFCPEGPSCKFMHPRFELPMGTTEQPP  180
            IKDCPWYDRGFCKHGPLCRHRHTRRVICVNYLVGFCPEGPSCKFMHPRFELPMGTTEQPP
Sbjct  121  IKDCPWYDRGFCKHGPLCRHRHTRRVICVNYLVGFCPEGPSCKFMHPRFELPMGTTEQPP  180

Query  181  LPQQTQPPTKQRAPQVIGVMQSQNSSAGNRGPRPLEQVTCYKCGEKGHYANRCTKGHLAF  240
            LPQQTQPP KQR PQVIGVMQSQNSSAGNRGPRPLEQVTCYKCGEKGHYANRCTKGHLAF
Sbjct  181  LPQQTQPPAKQRTPQVIGVMQSQNSSAGNRGPRPLEQVTCYKCGEKGHYANRCTKGHLAF  240

Query  241  LSGQ  244
            LSGQ
```
